# Leadership dynamics in musical groups: Quantifying effects of musical structure on directionality of influence in concert performance videos

**DOI:** 10.1371/journal.pone.0300663

**Published:** 2024-04-03

**Authors:** Sanket Rajeev Sabharwal, Matthew Breaden, Gualtiero Volpe, Antonio Camurri, Peter E. Keller

**Affiliations:** 1 DIBRIS, University of Genoa, Genoa, Italy; 2 MARCS Institute for Brain, Behaviour and Development, Western Sydney University, Penrith, Australia; 3 Center for Music in the Brain, Department of Clinical Medicine, Aarhus University & The Royal Academy of Music Aarhus, Aarhus, Aalborg, Denmark; Northumbria University, UNITED KINGDOM

## Abstract

Music ensemble performance provides an ecologically valid context for investigating leadership dynamics in small group interactions. Musical texture, specifically the relative salience of simultaneously sounding ensemble parts, is a feature that can potentially alter leadership dynamics by introducing hierarchical relationships between individual parts. The present study extended previous work on quantifying interpersonal coupling in musical ensembles by examining the relationship between musical texture and leader-follower relations, operationalised as directionality of influence between co-performers’ body motion in concert video recordings. It was hypothesised that the directionality of influence, indexed by Granger Causality, would be greater for ‘homophonic’ textures with a clear distinction between melody and accompaniment parts than for ‘polyphonic’ textures with less distinction between melody and accompaniment. This hypothesis was tested by using pose estimation algorithms to track instrumentalists’ body movements in a string quartet and a clarinet quintet, and then applying Granger Causality analysis to their head motion to estimate directional influence between instrumentalist pairs for sections of the pieces that varied in texture. It was found that Granger Causality values were generally higher (indicating greater directionality of influence) for homophonic than polyphonic textures. Furthermore, considering melody and accompaniment instrument roles revealed more evidence for the melody instrument influencing accompanying instruments than vice versa, plus a high degree of directionality among accompanying instruments, in homophonic textures. These observed patterns of directional information flow in co-performer body motion are consistent with changing leader-follower relations depending on hierarchical relations between ensemble parts in terms of the relative salience of melodic material in the musical texture. The finding that automatic pose estimation can detect modulations of leadership dynamics in standard video recordings under naturalistic performance conditions has implications for investigating interpersonal coordination in large-scale music video datasets representing different cultural traditions, and for exploring nonverbal communication in group activities more generally.

## Introduction

Interpersonal coordination and entrainment characterise a wide range of human interactions, from spontaneous audience applauding to strategic play in team sports and artistically significant collective displays in music and dance. Small ensembles of musicians provide a valuable and practical setting for investigating these phenomena from a variety of perspectives, such as biomechanical, computational, psychological, and neuroscientific [1–4]. Ensemble co-performers synchronise their body movements and sounds with high accuracy and flexibility to convey musical structure and emotive information to one another and the audience. This synchronisation, achieved through rigorous rehearsal and a shared understanding of musical intent, is critical for the successful delivery of a performance and the realisation of a collective musical expression [5–8]. The resulting musical communication is multimodal in the sense that visual information can play a role in live and recorded performances, even though auditory information is typically primary [9–12]. In live performances, the visual component not only facilitates tighter synchronisation among performers but also enriches the interaction with the audience. In recorded performances, visual cues continue to play a role, as seen in the widespread appreciation and engagement with music videos and live-recorded performances [13, 14].

In addition to body movements that directly trigger sounds, musicians produce movements that are not technically required for sound production, such as head nods and torso swaying, but contribute to regulating an individual’s performance while conveying musical structure, expressive intentions, and underlying musical meaning to others [9, 15, 16]. These movements may also constitute an embodiment of the musical intentions, where the physical gestures are intertwined with the sound modulations to enhance the musical expression. In musical ensembles, these ancillary movements, whether consciously intended or reflexive, serve as visual cues that bolster communication and aid co-performers in synchronising their actions. Visual cues become especially beneficial at points of heightened uncertainty (e.g., tempo changes or periods of silence) [17–19], complementing auditory information by providing continuous visual feedback that co-performers can utilize to anticipate each other’s sounds [20], across multiple timescales reflecting the hierarchical musical structure [21].

Coordination of sounds occurs on short timescales in the range of milliseconds, whereas body sway and other movements align over more extended periods corresponding to higher levels of musical structure, like phrases (i.e., organisational units perceived as coherent when presented in isolation, analogous to sentences in speech) [21–23]. Previous work has demonstrated that the coordination of ancillary body motion is systematically related to sound synchrony and ensemble cohesion [7, 23–25], suggesting that visual and auditory information provide parallel channels for musical communication [26]. Moreover, the coupling between performers in body movements and sounds is not static but dynamic and changes over time [27–29]. One aspect of interpersonal coupling that can vary is the degree to which it is symmetrical versus asymmetrical, in terms of the directionality of information exchange or influence among co-performers, which can reflect musical leadership relations [2, 4, 30, 31]. Here, we use the term ‘leadership’ broadly to refer to a spectrum of relations spanning no leader (as in bidirectional influence), a single leader and one or more followers, or multiple leaders and followers.

In many forms of collaborative joint action, individuals within small groups assume complementary roles, such as leader or follower, observable in tasks ranging from lifting a bulky item to collective dance [10, 32, 33]. In musical ensembles, explicit leadership roles can be strategically assigned or dictated by convention (e.g., the first violinist in a string quartet), whereas implicit roles can emerge spontaneously through constraints related to task structure or participant characteristics, including individual capabilities and personality [11, 31, 34–36].

Previous research has investigated leadership dynamics in musical ensembles using optical motion capture systems to track co-performers’ body motion. Early studies with piano duos demonstrated the sensitivity and reliability of this technique by showing that head movements of designated leaders precede those of followers [10] and that sound synchrony is high to the extent that the body sway motion of the pianist playing the melody part precedes motion of the accompanying pianist [7]. Related work with larger ensembles has investigated the directionality of information flow in co-performers’ motion by assessing Granger Causality (GC). GC is a statistical hypothesis test used to determine if one time series can predict another. Specifically, it evaluates whether past values of one time series (e.g., Musician 1’s motion) provide valuable information in predicting future values of another time series (e.g., Musician 2’s motion) better than using the past values of the second time series alone. This method thereby gives insights into potential causal relationships between the two time series [37].

A seminal study used GC to quantity information flow between the baton motion of conductors and bow motion of members of an orchestral string section [2]. This study revealed that the method was sufficiently sensitive to detect distinct levels of information flow for different conductors and counteracting effects where increased conductor influence was associated with decreased influence among instrumentalists. Additional evidence for the validity of using GC measures to index ensemble coordination was provided by the finding that the quality of performances was judged to be high to the extent that the conductor influenced the instrumentalists.

A subsequent study [38] measured GC between head movements of members of a string quartet while introducing ‘perturbations’ in the form of alterations of rhythm and dynamics (loudness) known only to the leader. Results indicated that the uni-directional influence of the leader was reduced, and mutual influence among co-performers increased during periods following the perturbations, especially when playing sections where coordination was challenging due to complex relations between parts in terms of rhythm, dynamics, or articulation. Consistent with the notion that leadership is especially pertinent to coordination challenges, a study focusing on head motion in duos consisting of a pianist and a clarinettist found greater directionality of coupling at the opening section of a piece and in a section characterised by rhythmic uncertainty [34]. Leader-follower relations are thus changeable rather than fixed. Flexibility in leadership was demonstrated in a study showing that secretly assigned leaders in a string quartet had a greater influence on followers’ body sway than vice versa and that assigning different members as leaders resulted in changes to their relative predictive influence on co-performers [27]. A positive correlation between the overall information flow and judgments of performance quality was also found. This reinforces the validity of GC as a measure of interdependencies in body motion and speaks to its relevance in gauging ensemble cohesion.

The findings of studies that applied GC analysis to ensemble motion capture data suggest that leadership dynamics can be reliably quantified based on directional analysis of body motion and can change flexibly in response to co-performers’ goals and musical structure. Musical texture is a structural aspect linked to variations in interpersonal coupling in ensemble performance [27–29]. In music, ‘texture’ denotes the layering of instrumental parts and their relationships, which can often manifest in terms of the relative saliency of parts and the intricacy of their pitch and rhythmic relations [39]. Homophonic textures are characterised by a strong vertical harmonic relationship between parts, with a clear distinction between melody and accompaniment [34, 36, 38, 40]. In polyphonic music, by contrast, multiple instruments or voices play different melodies at the same time, giving each musician some independence and encouraging the listener to shift the focus of attention horizontally the flow of the multiple melodies [31, 41–43].

A prior study by Sabharwal et al. [44] examined the effects of texture on interpersonal coupling in small musical groups by analysing the body motion of instrumentalists in videoed concert performances of ensemble pieces that contained homophonic and polyphonic sections. This study involved developing a computational framework and system for automatic pose estimation (huSync) to analyse body motion in video recordings as an alternative to marker-based motion capture methods, allowing concert performances to be studied conveniently in an ecological setting. The pose estimation-based system extracted full-body motion data for each performer in a string quartet (consisting of two violins, viola, and cello) and a clarinet quintet (a string quartet with added clarinettist). Then, phase relations between the head motion trajectories were measured for all possible pairs of performers. Comparing the results across textures revealed that phase coupling of co-performers’ head motion was generally stronger for polyphonic textures (where leadership is ambiguous) than for homophonic textures (where there is a clear melodic leader).

Ambiguous or changeable leadership in polyphonic textures thus encourages uniformly high interpersonal coupling. In contrast, distinct leader-follower roles in homophonic textures engender lower overall synchrony due to potential asymmetries in coupling. This interpretation is consistent with laboratory studies showing that dyadic coordination can be less precise when leader-follower roles are designated than when they are not [45–47], possibly because the lack of leadership assignment facilitates efficient mutual adaptation and anticipation [48]. However, our previous study did not include directional measures of interpersonal coupling. Therefore, whether the observed differences between homophonic and polyphonic textures were attributable to different leadership dynamics remained to be determined.

The present study addresses this question by examining the relationship between musical texture and leader-follower roles based on assessment of the directionality of interpersonal coupling between ensemble co-performers. To this end, we extended the huSync computational framework and pose estimation-based system for analysing body motion developed by Sabharwal et al. [44] to compute GC measures between the head motion of pairs of instrumentalists in the videoed concert performances. Based on previous work [2, 38], it was assumed that statistically significant GC tests, which can be computed in both directions within instrumentalist pairs, indicate the degree to which each performer exerts influence on the other performer within a pair. Our general hypothesis was that interpersonal coupling is more strongly directional when a clear leadership hierarchy exists and, therefore, that GC tests will yield more substantial evidence for directional coupling for homophonic than polyphonic textures. Furthermore, we expected that the role of the performer (melody vs. accompaniment) would modulate the directionality of influence in homophonic textures. Specifically, we hypothesised that GC tests would more often yield evidence that the performer playing the melody influenced the other performers than vice versa. We did not have an a priori hypothesis about the directionality of influence among accompanying players in homophonic textures. We employed linear mixed-effects models (LMM), Analysis of Variance (ANOVA), and Generalised Linear Mixed Model (GLMM) analyses to assess the effects of musical texture on GC values and the directionality of influence, with results corroborated across methods for robustness and reliability.

## Materials and methods

### Dataset

The dataset consisted of videos of performances of two musical pieces featured in a concert held in 2017 by the Omega Ensemble, a professional chamber music group from Australia. One piece was the Clarinet Quintet in B minor (Op. 115) written by Johannes Brahms (referred to as Quintet), and the second piece was String Quartet No. 1 in A major written by Alexander Borodin (referred to as Quartet). The Quartet is scored for violin 1, violin 2, viola, and cello. The Quintet uses the same string instruments plus a clarinet. The duration of the Quartet performance was 39 minutes and 13 seconds, while the duration of the Quintet performance was 40 minutes and 38 seconds. Both pieces contain four movements with contrasting musical characters and textural variations that enable the selection of equal numbers of homophonic and polyphonic sections of similar duration. Approval of all ethical and experimental procedures and protocols was granted by the Human Research Ethics Committee at Western Sydney University (protocol number H10487) and performed in line with the Declaration of Helsinki. Members of the Omega Ensemble were recruited in 2017. They provided written informed consent to participate in the study in the knowledge that individual participants would be identifiable because the data consisted of video recordings. A previous study using these videos [44] demonstrated that they are suitable for investigating the effects of textural variations on interpersonal coordination in musical ensemble performances from a natural concert setting. However, while our earlier analysis focused on a global measure of interpersonal coupling (phase-locking values), the current study focuses specifically on the directionality of coupling (Granger Causality).

Videos of the performances were recorded using a Canon 1DX camera body and a Canon EF 70–200 1:2.8 L zoom lens as QuickTime movies (.MOV) with dimensions 1920 × 1080 pixels at 25 frames per second. The full videos were split into parts based on textural annotations (homophonic and polyphonic) that were made in ELAN [49] based on musicological analysis of the published scores by author MB, which were checked by author PEK (both authors have university qualifications in musicology). We operationally defined musical phrases as sections of the pieces that were made up of coherent thematic material presented in a consistent texture. Alterations in both the thematic content and texture serve as phrase boundaries. Although the resulting phrase units are sometimes longer than those typically used in musicological analysis, they provide more suitable units for investigating how structural change affects the directionality of influence.

**Fig 1:**
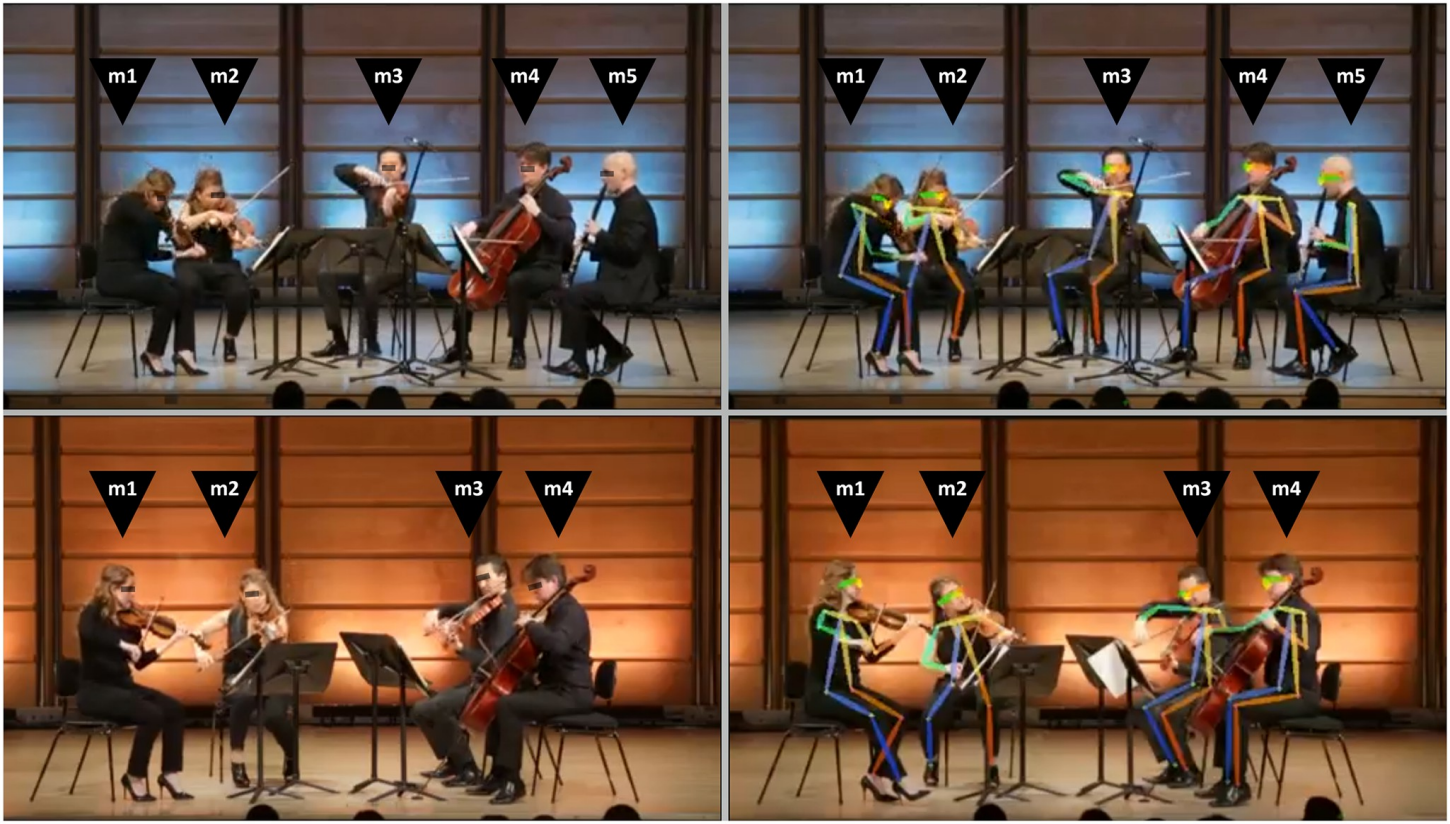
Images from the performance of the Quintet (Top Left) and the Quartet (Bottom Left), along with the outputs available with tracked key points using a pose estimation algorithm (Top Right and Bottom Right). The arrangement of musicians from left to right are labelled as m1 to m4 (Quartet: m1—violin1, m2—violin2, m3—viola, m4—cello) and m1 to m5 (Quintet: m1—violin1, m2—violin2, m3—viola, m4—cello, m5—clarinet).

For each phrase, textural categorisation, the total number of performing instruments, and instrument roles (such as melody, counter melody, or harmonic accompaniment) were annotated in different layers within the ELAN interface. Data from each layer of the annotated ELAN file for each piece was extracted to extract video timecodes for each phrase and associated textural categorisation. The bar numbers from the score corresponding to each excerpt are provided in S1 Table (For Quintet: Brahms, J. (1892). Clarinet Quintet, Op. 115. N. Simrock.) and S2 Table (For Quartet: Borodin, A. (1884)). String Quartet No. 1. Hamburg: D. Rahter.). Based on this information, phrases that had consistent texture and predominantly all instruments playing throughout were selected for analysis. In Table 1, we present the selected phrases used for analysis in our study, and Fig 1 illustrates the specific positioning of musicians, arranged from left to right, along with appropriate labels. We made use of an equal number of homophonic and polyphonic phrases to have balanced textural classifications and similar durations (ranging from 15 to 38 s).

**Table 1:** Summary statistics for all texturally consistent phrases in the Quintet and Quartet and the subset selected for analysis based on matching phrase duration.

		Full Dataset Duration (s) and Count					Selected Phrases Duration (s) and Count				
Piece	Texture	Min	Max	Med	Avg	Count	Min	Max	Med	Avg	Count
Brahms	Homophonic	15.03	38.20	19.74	21.57	27	16.16	38.20	21.60	23.74	12
Brahms	Polyphonic	15.49	33.08	23.10	23.53	20	15.49	27.55	20.16	21.11	12
Borodin	Homophonic	15.30	24.97	18.32	19.12	10	15.30	24.97	18.32	19.12	10
Borodin	Polyphonic	15.14	29.63	21.27	21.01	11	15.14	29.63	20.92	20.80	10

### Computational framework and procedure

**Fig 2:**
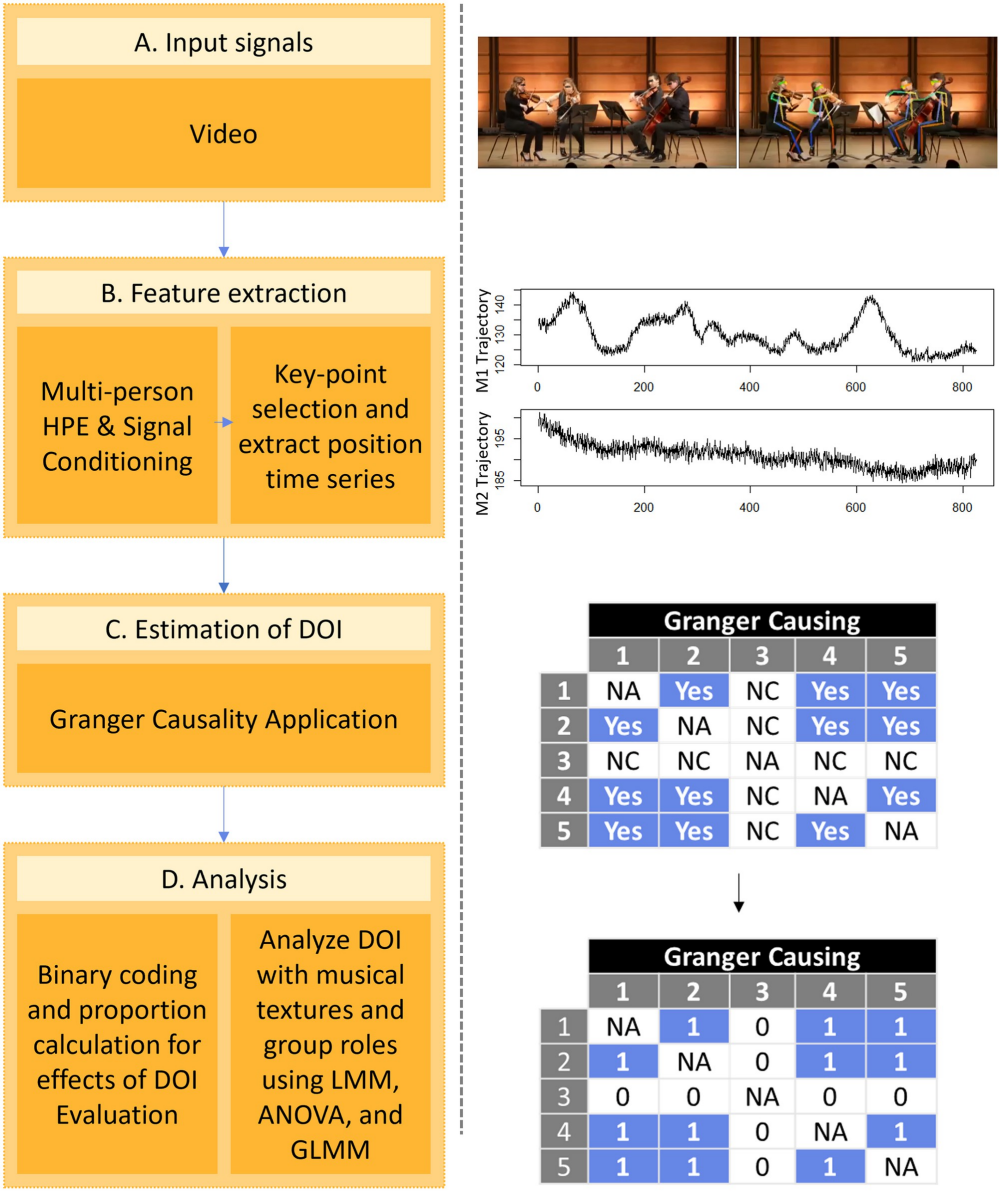
An illustration of the huSync computational framework and its system architecture as implemented in our study, showing the transition from video input signals to statistical analyses. The accompanying images on the right delineate critical phases of the workflow and its intrinsic procedures.

The motion data of each performer in the video recordings were extracted through a customised processing pipeline. Our previous study [44] developed the huSync computational framework utilised by this pipeline, which has been updated to include a new experimental and analytical approach that addresses the current research questions about information flow directionality. Fig 2 provides an overview of our approach, which includes four blocks, and is based on a well-established system for analysing body movements and gestures that convey expressiveness [50, 51]. The code and data pertinent to this study are openly accessible on Github. The repository contains fully commented code and detailed instructions to facilitate straightforward reproducibility. Additionally, the original huSync code is publicly accessible on Github [44].

Our process was organised into a structured funnel of steps as follows:

1. After selecting videos based on our analysis criteria (see Table 1), we organised the recordings of the chosen phrases and readied them to be passed onto the second phase for feature extraction (see Fig 2(A)).
2. We use AlphaPose [52], a multi-person pose estimation algorithm to extracted motion and postural data from the chosen video recordings (see Fig 2(B)). The algorithm generates a JSON file containing the trajectory information of body parts sampled at 30 fps, with the nose key point serving as the best available representation of the head. We obtain kinematic information as a position time-series data for further analysis using the nose key point. We then used the data in the next step to quantify the directionality of influence among performers. To ensure the data are stationary, we applied first-order differencing to the log-transformed time series, a standard practice based on previous studies [53–55].
3. We employed the *grangertest* function from the lmtest package [56] in R Studio to evaluate the directionality of information flow. The function measures the predictive relationship between two time series (see Fig 2(C)). In our analysis, ‘X’ and ‘Y’ symbolise the time series of movements for any two given performers, respectively, and ‘order’ denotes the number of lags incorporated in the model (typically set to 1 by default). To illustrate, consider two arbitrarily chosen performers labeled as J and K; we utilised the *grangertest* function to investigate the extent to which the movement of performer J (X) predicts the movement of performer K (Y), and vice versa (X → Y and Y → X). This method allowed us to assess the presence of Granger causality within the pair’s interactions. It is important to note that our measurements within the video frame’s coordinates offer a parallel to each performer’s bodily movements due to the camera’s fixed orientation relative to the group. This means that the horizontal axis of our video analysis closely matches the natural horizontal axis of the performers’ body movements, thus serving as an approximate measure for lateral head movements. If the p-value yielded by the Granger test was less than .05 (our criterion for statistical significance), we rejected the null hypothesis and inferred a statistically significant predictive relationship between the two time series in the specified direction. To account for the delay between stimulus and response that is common in musical performances [27, 57], as well as in most behaviours, we performed GC tests for a lag of up to 1 second, setting the order to 30 (equal to the sampling rate) to test for multiple lag-lengths, and examine the nose key-point separately for each pair in both possible test directions (X → Y and Y → X). We extracted the GC values, including F and p values from each Granger test, and recorded the binary numbers [0, 1] indicating the outcome of each test. A value of 1 indicates a statistically significant predictive relationship (assumed to indicate influence or information flow) between the performers in the tested direction, while 0 indicates the absence of a significant predictive relationship in that direction. When GC tests in both directions were statistically significant for a given instrumentalist pair, a ‘1’ was coded for both directions. The main analyses were based on the proportion of significant GC tests among pairs of musicians for each phrase. Specifically, binary values indicating whether (1) or not (0) each Granger test was significant were averaged across all pairs of performers (separately for each test direction, X → Y and Y → X) for each phrase representing the two textures for each piece. These proportion data were then passed onto the next step to analyse the directionality of influence among co-performers to address our research questions.
4. In this stage (see Fig 2(D)), we carried out several statistical analyses on the GC values we obtained to address our hypothesis on the directionality of influence. We recorded results for all possible pairs of instrumentalists for each musical phrase. Note that the aim of these analyses was not to determine whether interaction is taking place at greater than chance levels (given that expert ensembles were intentionally coordinating highly rehearsed performances in a public concert setting), or whether GC can capture leader-follower relations in ensembles (since this has been previously demonstrated in a number of studies). Our question instead concerns the quality of interaction, specifically related to leadership relations, and whether these relations vary across conditions in relative terms. The results for all pairs for the Quintet can be found in S3 Table and those for the Quartet in S4 Table. In R, we conducted two primary statistical analyses [58, 59], one using a linear mixed-effects model (LMM) to test for general effects of musical texture on GC values, followed by an Analysis of Variance (ANOVA) to address directionality of influence effects related explicitly to melodic leadership. Musical piece (Brahms Quintet and Borodin Quartet) was included as a random effect in these analyses since we did not have hypotheses regarding the pieces (they comprised a convenience sample that were on the ensemble’s program at the time of data collection), but rather were interested effects that generalise beyond them. Shapiro-Wilk tests indicated that proportion data were not normally distributed in some conditions, even following arcsine transformation. Therefore, we report analyses on untransformed data (additional analyses with arcsin-transformed data yielded similar results). However, given these violations of the normality assumption, we also conducted binomial Generalised Linear Mixed Model (GLMM) analyses on raw binary GC values to check whether equivalent effects are obtained. Obtaining consistent outcomes for the LMM and GLMM analyses would provide evidence for the robustness and reliability of results. Such consistency was observed, and we only report the LMM and ANOVA results in the article (because these tests are standard and facilitate comparison with other studies in the literature). Due to the large number of GC tests run per musical excerpt (to assess exhaustive pairwise relations between instrumentalists), we addressed the issue of potential false positives by correcting for multiple comparisons using the Bonferroni method in supplementary analyses reported in S1 Appendix (see S1 Appendix). The results were overall consistent with those reported here.

## Results

**Fig 3:**
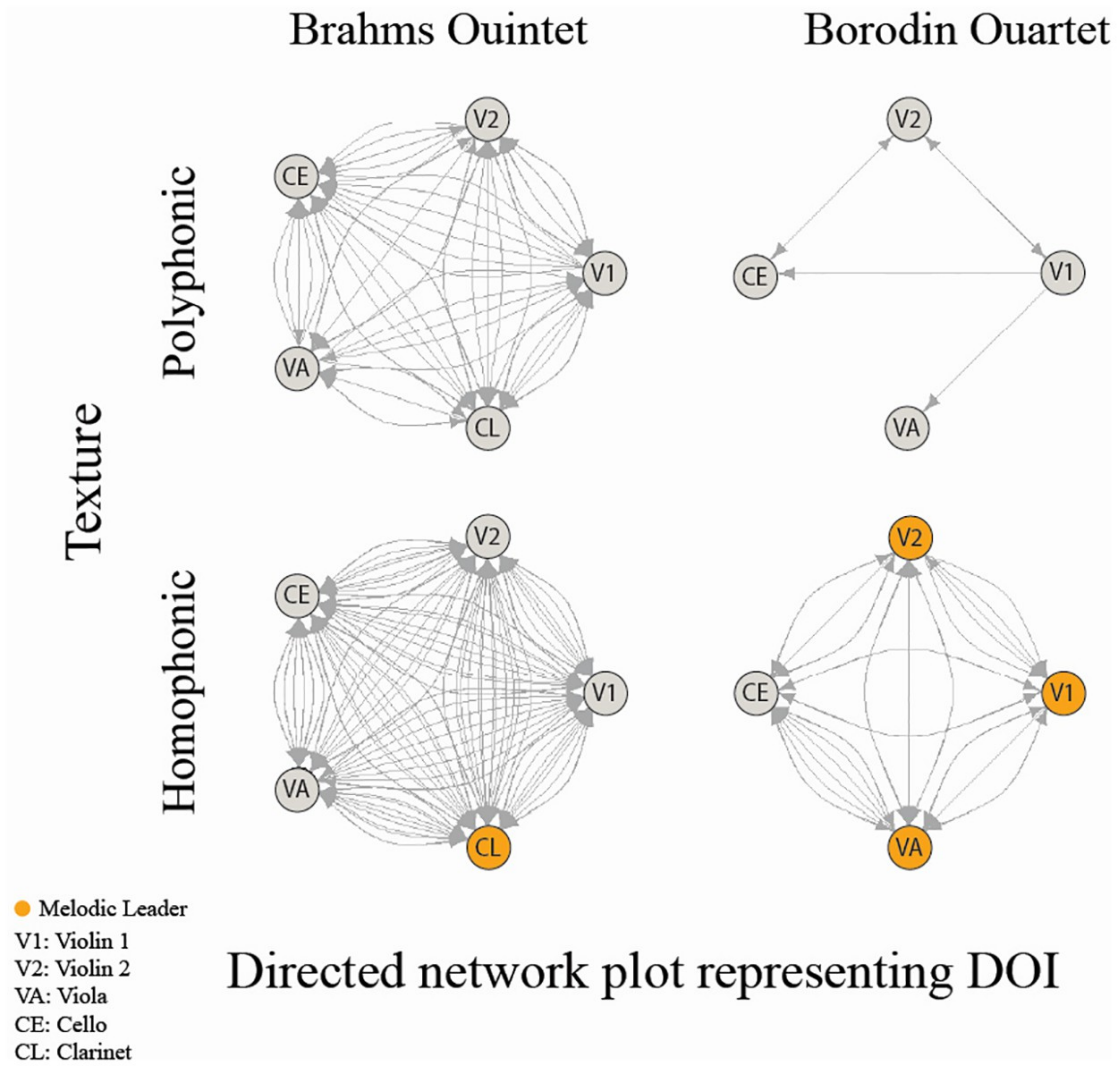
Directed network plots for ensemble GC values by instrument for each texture in Quintet and Quartet musical pieces, representing the directionality of influence (DOI). Edge direction indicates DOI across all phrases. Homophonic textures have a clear melodic leader, while leadership is assumed to be distributed in polyphonic textures. Each node represents an individual instrumentalist, and the yellow dot indicates the melody instruments (which varied across the analysed phrases for the Quartet) in homophonic textures.

Fig 3 shows network plots of GC values representing statistically significant directional linkages for individual instrument pairings as a function of musical texture in the Quintet and Quartet performances. Visual inspection of the plots reveals that connections are denser for the Quintet than the Quartet. This effect was not of interest in the present study, since musical piece was considered as a random effect in our analyses. Note that comparing the pieces would be problematic and inconclusive because they vary on multiple confounding parameters (e.g., number of players, composer, tempi, key, and stylistic elements).

Of note, it is also evident that there is a greater density of connections for homophonic than polyphonic textures, consistent with our main hypothesis that interpersonal coupling would generally exhibit higher directionality in homophonic textures with a clear melodic leader compared to polyphonic textures with distributed or changing leadership roles. Evidence for or against our additional specific hypothesis that the melody player would influence other players more than vice versa in homophonic textures is less readily discernable from visual inspection of these network plots alone since numerous connections exist not just between the melody player and other instrumentalists, but also among these other players. This underscores the fact that our observations are grounded in a probabilistic framework, reflecting tendencies not certainties. For example, while findings suggest a melody player often assumes a leadership role, it does not rule out influential interactions from other ensemble members. Separate statistical analyses on the GC values were conducted to address each of the two hypotheses.

### General effect of musical texture

**Fig 4:**
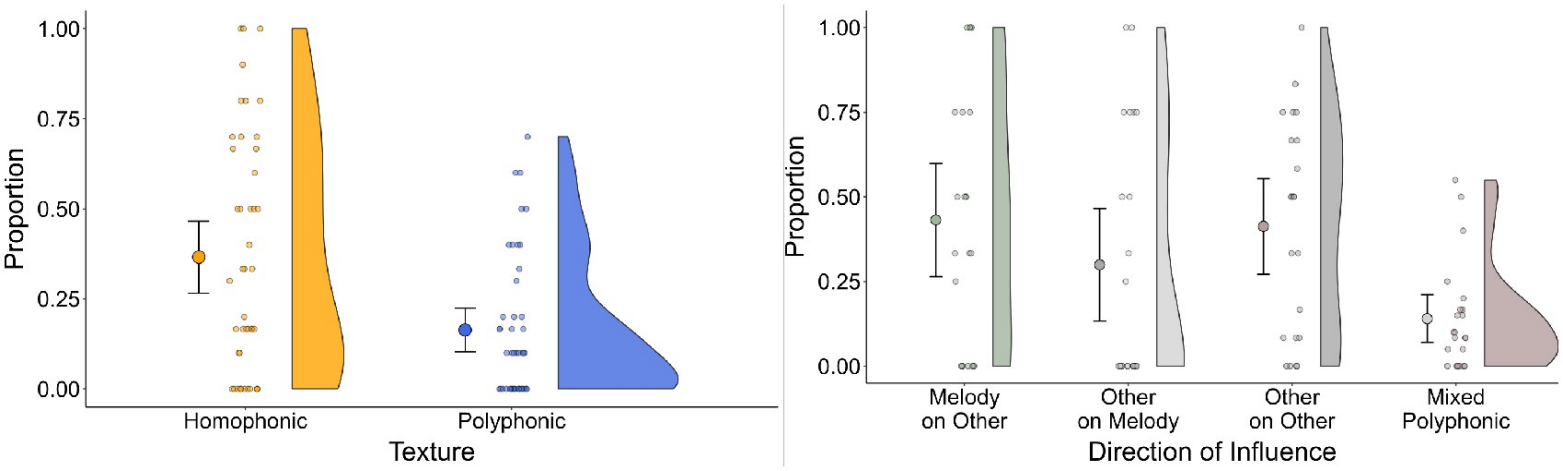
(A—Left) Proportion of significant GC values for homophonic and polyphonic textures, averaged across the Quintet and Quartet; (B—Right) Proportion of significant GC values for four categories of musical roles adopted by co-performers, indicating the directionality of influence in the musical ensemble.

The hypothesis that interpersonal coupling would exhibit higher directionality in homophonic textures than polyphonic textures was tested by examining the proportion of instrumentalist pairs exhibiting statistically significant GC test values as a function of musical texture. The results in Fig 4(A) revealed a higher proportion of significant GC values for homophonic compared to polyphonic textures, thus confirming the hypothesis. To further evaluate this effect, we computed a LMM using the lme4 package in R [60], with texture as a fixed factor and piece, part, phrase, and direction of the GC test (within each pair of instrumentalists) as random effects. We included the piece as a random effect because our hypotheses were not specific to the two particular musical works featured but rather representative of Western chamber music in general.

A likelihood-ratio test indicated that the full model with texture provided a better fit to the data than a reduced model, including only the random effects (*χ*^2^(1) = 10.90, *p* <.001; Log Likelihood = 17.20 (full) vs 11.80 (reduced), AIC = -20.40 vs -11.50, BIC = -3.10 vs 3.37). The full model revealed a statistically significant effect of texture on GC values (Effect Estimate = -0.248, SE = 0.070, *t* = -3.54, *p* = 0.001, 95% CI [-0.388, -0.107]), indicating that the directionality of coupling was reliably higher in homophonic than polyphonic textures.

### Specific effects of musical roles

The second analysis tested the hypothesis that the instrument playing the melody would exert greater influence on instrumentalists playing accompaniment material more than vice versa in homophonic textures. For this analysis, we classified the GC test outcomes based on the musical roles of the instrumentalists, which included melody, accompanying (other), or mixed (in polyphonic settings). We identified four categories of direction of influence, which can be seen in Fig 4(B): (1) melody instrument influencing accompanying instruments (Melody on Other), (2) accompanying instruments influencing the melody instrument (Other on Melody), (3) accompanying instruments influencing other accompanying instruments (Other on Other) in homophonic textures, and (4) mixed roles in polyphonic textures. GC values were entered into a LMM with the direction of influence category as a fixed factor and piece, part, and phrase as random effects. This full model provided a better fit to the data than a reduced model with only random effects, as indicated by a likelihood-ratio test (*χ*^2^(3) = 15.50, *p* < .01; Log Likelihood = -1.06 (full) vs -8.82 (reduced), AIC = 18.10 vs 27.60, BIC = 37.90 vs 40.00).

We conducted a follow-up ANOVA with three planned orthogonal contrasts to determine the specific effects of the direction of influence. These contrasts compared homophonic leadership categories combined (Melody on Other, Other on Melody, and Other on Other) versus the mixed polyphonic category (which is equivalent to the analysis reported above, but with different degrees of freedom), homophonic categories including a melody player (Melody on Other and Other on Melody) versus the homophonic category without a melody player (Other on Other), and the category reflecting melody instrument influence on other instruments (Melody on Other) versus accompanying instrument influence on the melody instrument (Other on Melody). This analysis indicated that GC values were significantly higher for homophonic than polyphonic textures *t*(45.1) = 2.849, *p* = .0066, 95% CI [0.212, 1.235] and for melody instrument influence on others than for other instrument influence on the melody instrument *t*(46.8) = 2.520, *p* = .015, 95% CI [0.027, 0.238]. This latter finding is consistent with our specific hypothesis about musical role. In addition, we found that GC values for homophonic pairings including a melody player were not significantly different from values for homophonic pairings without a melody player *t*(46.8) = −1.039, *p* = .304, 95% CI [-0.278, 0.089]. This post-hoc finding was not hypothesised but is informative to the extent that it indicates high directionality of influence among accompanying instrumentalists.

## Discussion

The present study investigated the effects of variations in musical texture on leadership dynamics in musical ensembles by using pose estimation techniques and Granger Causality (GC) measures to assess the directionality of interpersonal coupling between instrumentalists in videos of concert performances by a string quartet and a clarinet quintet. Our main finding was that the proportion of significant GC values, representing the directional influence of head motion in pairs of co-performers, was higher for homophonic textures, where there is a clear distinction between melody and accompanying parts, compared to polyphonic textures, where melodic content is distributed across parts. This finding supports the general hypothesis that the distinction between parts in homophonic textures is associated with leader-follower relations reflected in higher directionality in interpersonal coupling. Furthermore, our analyses revealed that the melody instrument had a greater influence on other instruments than vice versa in homophonic textures, consistent with the assumption that the melody player serves as the leader. Our results extend our previous findings on non-directional coupling in the same performances [44] and build on previous studies that used GC to assess leadership dynamics in other ensemble settings [2, 17, 27, 38, 40, 61] by highlighting specific effects of musical texture as well as demonstrating the viability of quantifying leader-follower relations from patterns of body motion extracted from naturalistic video recordings.

### Links between musical texture and leadership

Regarding the effects of musical texture, finding greater directionality in coupling for homophonic textures, with information flowing from melody player to accompanying players more than vice versa, sheds light on our previous result that phase coupling was generally weaker in homophonic than polyphonic textures [44]. We previously argued that this finding could be due to coupling being more evenly distributed across all performers in polyphonic textures, whereas accompanying performers are mainly coupled to a single melodic leader in homophonic textures. Present results are generally consistent with interpretation, though the finding of directional influence among accompanying players in homophonic textures adds further nuance to the picture.

Overall, our present results suggest that coupling may be relaxed when leader-follower relations are demanded by musical structure. Such relaxation may enable leader-follower roles characterised by asymmetries in the degree to which co-performers engage in temporal adaptation and anticipation. During rhythmic interpersonal coordination, temporal adaptation processes keep interpersonal asynchronies in check by implementing reactive error correction and anticipatory processes to enable the prediction of upcoming event timing [31, 62]. Previous work on leader-follower timing suggests that greater adaptation and anticipation is observed in the follower than the leader at the level of instrumental movements [11, 36, 63, 64], and our results show that such asymmetries may generalise to ancillary body motion. Other movement features that could additionally have contributed to the present results include higher complexity of motion or greater need for self-regulation in polyphonic textures, similarly explored in studies probing effects of coordination challenges on ancillary motion [7, 34, 65]. While further investigation is necessary to confirm this using appropriate analytical techniques, the importance of body motion cues indicates that musical communication might engage coordination across multiple timescales and sensory modalities [30]. This engagement could potentially share characteristics with the multimodal nature of diverse communicative signals, as suggested by prior research [66, 67].

Questions remain about whether the effects of musical texture on leadership dynamics are affected by other aspects of musical structure, such as phrase position. Our previous study found that the effect of texture varied depending on position within the musical phrase. Coupling was stronger for polyphonic than homophonic textures at the beginning and middle portions of phrases but then decreased to similar levels at phrase endings. It was argued that leader-follower relations were adopted at phrase endings in polyphonic textures due to coordination challenges at these points. The tempo tends to slow down at phrase endings and successive phrases can be separated by silent pauses of variable duration [31]. Previous research has shown that the resulting uncertainty at phrase boundaries triggers increased communicative behavior, such as eye gaze and larger amplitude motion [17, 34, 68]. Related work has shown that leaders tend to make their timing more predictable by increasing the timing regularly and/or the amplitude of their movements [6, 10, 45, 69]. These behavioural modifications are instances of a more general phenomenon of coordination smoothers [70]. Based on these previous findings, direct influence from the melody player to accompanying players could increase at phrase endings. However, we did not investigate the effects of phrase position in the current study due to issues of time series length for reliable GC estimates (see Limitations and future work). Nevertheless, future work could investigate such effects with experimental manipulations applied over more extended musical sections (e.g., by using specially composed pieces) where coordination challenges are systematically varied. Examining patterns of information flow among accompanying players could be especially revealing in this context [71], given our finding that such interpersonal dynamics are present and measurable.

Another issue relevant to the mechanisms underlying the link between musical texture and leadership is whether the hierarchical distinction between melody and accompanying parts in homophonic textures automatically induces leader-follower relations. Previous work with piano duos has shown melody lead in keystroke asynchronies and body sway [7, 10], but the degree to which this emerges spontaneously or as a deliberate strategy still needs to be determined. The finding that leader-follower relations are more consistent at the level of keystrokes than body sway [7] suggests that the latter may serve strategic purposes or vary in order to display different expressive intentions. Relatedly, it has been found that the perception of auditory leader-follower relations, specifically the temporal lag of sound in accompanying versus melody parts, is susceptible to bottom-up effects of auditory streaming, indicating automatic processing [72], while head motion is sensitive to leadership instructions, suggesting strategic use of communicative ancillary movements [27]. In the present study, leadership was not explicitly instructed, which suggests that co-performers negotiated leadership based on structural constraints in the music. At the same time, the nature of such negotiation has proven difficult to pin down in laboratory studies [73]. Analysing verbal communication between group members during rehearsals in natural settings [74] is a promising future avenue to address such negotiation.

Regardless of whether leader-follower relations emerge spontaneously or through expressed intentions, attention plays a pivotal role in directing information flow. Ensemble performers, when employing “prioritised integrative attending,” balance the distribution of attention between their own parts and those of their co-performers, while also monitoring the overall ensemble sound [39]. Despite one’s part presumably being the highest priority [75] in the case of homophonic textures, accompanying players may allocate attentional resources disproportionally toward the melody part, as well as to parts played by other accompanying players. The melody player, while also emphasising their own part, might be receptive to these dynamics. These attentional strategies, whether intentionally implemented or automatically emergent, could influence interpersonal coupling directionally, given the established links between attention and sensory-motor coupling [31, 76, 77]. The bidirectional adjustments intrinsic to ensemble settings further add to the complexity of these interactions.

### Benefits of studying natural coordination

Our study adds to the growing literature showing that body movement, including head motion, provides an valid metric to investigate interpersonal coordination and leadership dynamics in group settings [2, 27, 30]. Moreover, we demonstrate the potential of markerless motion capture technology to analyse such leader-follower dynamics in videos of music ensemble performances recorded during live concerts. Studying such performances is informative as they offer a naturalistic setting for musical communication. While this context presents an ecologically valid representation of ensemble dynamics, it may not be the only approach to investigating leadership in musical performances. Research on music and dance underscores the influence of performing “in situ” on communication quality. Factors like acoustics and audience presence can impact performers’ levels of motivation, attention, and arousal [78–80]. The nonverbal communication of emotions is a specific aspect that may benefit under these conditions, and the degree of enhancement may be reflected in increased information flow. Evidence for this link is seen in a study that found greater information flow in body sway when a trio were instructed to perform pieces with emotional expression than without emotion [81]. Heightened expressive intensity may, therefore, be associated with greater amplitude movements [82, 83] and the transmission of these cues between co-performers.

An additional advantage of using conventional video of ensemble performances is that it widens the potential pool of materials for analysis. Video-based analysis allows performances of other cultures to be studied when more specialised motion capture setups are not feasible [1], and therefore has the potential to overcome the prevailing WEIRD (Western, Educated, Industrialised, Rich, and Democratic societies) focus of research in psychology and neuroscience and subdisciplines such as music science [84]. A related practical benefit is that video is relatively neutral regarding group size, notwithstanding the issue of occlusion in large groups [44], and thus may help to accelerate the trend in the field to go beyond dyadic coordination to study groups of three or more performers [85]. An advantage of this upscaling, highlighted by the current results, is that it allowed us to examine the coupling between instrumentalists playing accompanying parts and interactions between melody and accompaniment. Our finding that the “other on other” influence was almost as strong as the “melody on other” influence in homophonic textures is noteworthy to the extent it captures the interaction between accompanying players. This finding suggests that to understand musical group dynamics, it is important to consider the interconnected network of the entire ensemble, in which subsets of performers function with some degree of independence [86]. Such independence is consistent with claims that it is necessary to balance the integration and segregation of psychological representations of “self” and “other” in ensemble performance [87–89], with the added nuance that there may be relatively high segregation between melody and accompaniment players, but a high degree of integration among the accompaniment players.

A further benefit of the current approach is conceptual. The observed effects of musical texture on leadership dynamics were not the result of an explicit experimental manipulation, nor were they post-hoc or data-driven findings. While these alternative approaches have particular strengths, experimental methods can lack ecological validity and data-driven findings can be challenging to interpret due to multiple possible contributing factors. Instead, our approach of segmenting the videoed performances based on musicological analysis of musical structure presents a middle ground that balances ecological naturalness and experimental control considerations. This feature highlights the benefit of using ensemble music from notated traditions, where the score functions as a script that constrains the actions of each performer while maintaining a degree of freedom for individual expression, as a domain to study the psychological dynamics of social interaction [3, 4].

### Limitations and future work

While markerless motion analysis offers significant advantages, it also presents challenges, such as alterations in frame resolution, occlusion, and lighting conditions. Comparatively, marker-based systems yield less noisy data than their markerless counterparts despite their higher costs and potential for causing participant discomfort. This quality disparity might narrow the scope of research questions that can be effectively addressed using markerless techniques. However, these techniques provide broader opportunities for studying group interaction, offsetting their limitations to a certain extent. This trade-off is an important consideration in the choice between selecting marker-based and markerless motion analysis methods.

The huSync system, as currently designed, does not differentiate between lateral (side-to-side) and vertical (up-down) head movements. It leverages input from a multi-person human pose estimation (HPE) algorithm, and, based on the musicians’ arrangement within the scene, it solely utilises position time series along the x-axis (horizontal). However, with advancements in the HPE domain enabling data extraction in 3D space, there is potential for enhanced analysis by capturing more nuanced motion trajectories of participants in multiple directions. Research findings indicate that different types of head motion play different functional roles in ensemble music performance, exemplified by head nods being linked to timekeeping [34, 90]. Given this, it may be possible to enhance the performance analysis capabilities of future versions of HuSync by distinguishing between up-down and side-to-side head motion in relation to ensemble dynamics. The current version of HuSync also does not account for the encoding of emotional intent through head movements [91, 92]. Incorporating this aspect could provide a more nuanced understanding of interpersonal coordination in ensemble settings. It should nevertheless be acknowledged that, although we foreground head movements, they are not the exclusive, nor necessarily the paramount, metric for synchronisation in ensemble contexts.

While the current study and previous work demonstrate the capability of using GC with body motion data in providing insights on ensemble leadership dynamics, the relationship of these measures to the coordination of sounds warrants further attention. We did not include measures of auditory communication via musical sounds in our analysis, and therefore our measures focus exclusively on visual nonverbal communication exhibited by ancillary motion. Previous research has established close relations between ensemble coordination at the level of sounds and body motion [6, 7, 23] and our previous work found correspondences between overall synchrony and audio event density [44]. Although signal processing techniques exist for separating sound sources from mixed recordings [93, 94], and recent work has assessed ensemble synchrony directly from the overall group output using recurrence quantification analysis [95], the types of recordings we analysed are not well-suited for these techniques. Sound source separation can be problematic to the extent that instrumental timbres are similar (as is the case of the string sounds in our dataset). The recurrence-based technique has to date been used to distinguish between coordinated and deliberately uncoordinated sections of a musical piece (where the composer’s score instructs the performers to play at random in the uncoordinated section), rather than subtle differences in coordination, let alone leader-follower relations. An alternative viable avenue for future work is to investigate how listeners and observers perceive the directionality of effect, as has been done with overall measures of group coordination [2, 27, 65].

Another issue to address relates to the fact that the present study focused on naturalistic ensemble performance, and did not compare co-performers’ body motion to movement patterns during solo performance. Previous research has highlighted the relevance of body movements in solo performance for regulating performance and communicating musical structure and expression [82, 96]. Incorporating a solo performance condition in future studies could provide a baseline for understanding how these functions are fulfilled for different musical textures in ensemble settings [9, 97]. It also bears mention that we did not directly ascertain distinct leadership roles from the musicians but inferred them from the intrinsic musical structure. This means that related patterns of body motion could be influenced by technical demands related to coordination. A potential avenue for subsequent research is examining the extent to which homophonic textures allow for more liberal head movements due to diminished synchronisation demands.

A further limitation is that, for technical reasons (see Links between musical texture and leadership), we did not address the potential effects of position within phrases on leadership dynamics. GC assumes that time series data are stationary (i.e., free from the drift that makes summary statistics vary over time). A standard method of dealing with potential non-stationarity, higher-order differencing [53, 55], can result in the loss of information regarding serial dependencies and a reduction in sensitivity to directional information flow. Such losses may be disproportionately impactful in short time series when introducing lags to maximise the likelihood of capturing delays in information flow associated with leader-follower relations. Although it is justifiable to apply GC to short time series in contexts where there is no alternative, such as non-human animal communicative signalling, and when done in parallel with complementary methods [98], in our view, the appropriateness of doing so for human musical interaction requires further investigation. Ultimately, it will be beneficial to explore alternative metrics and modelling techniques to capture dynamics of the strength and directionality of coupling in musical groups [86, 99, 100].

Another limitation relates to sample size. Our study focuses on members of a professional ensemble performing in two constellations, a string quartet and a clarinet quintet. It is therefore best considered a proof-of-principle case study that takes advantage of the availability of data from a top international ensemble. This raises questions about the generality of findings and, in particular, how the effects of musical texture on leadership dynamics might be affected by factors such as the particular musical piece, as well as musical expertise and familiarity with co-performers. Effects of musical piece were not analysed in the current study because the quartet and quintet varied on a range of stylistic parameters beyond the number of performers involved. Examining these factors systematically across a series of studies will be necessary to disentangle their effects in future work.

Regarding familiarity, a study of piano duos found that familiarity with a co-performer part facilitates the coordination of ancillary head motion and body sway, however, not necessarily instrument keystrokes [23]. Another study found that incongruent performance goals (concerning tempo changes) were resolved with rehearsal via stylistic assimilation [73]. However, that study did not resolve how leader-follower dynamics played a role in this process. There is evidence that such relations may become less relevant with increasing familiarity. An analysis of repeat performances by a string quartet [71] revealed that, as co-performers converged on a common stylistic interpretation and their body sway became more similar across repeats, the overall degree of information flow (assessed by GC) decreased, possibly because performers came to rely on feedforward processes driven by internal models rather than actual feedback [42, 89, 101]. Our findings suggest that in future work, it would be worthwhile to examine overall information flow and the specific directionality of information flow under such conditions as a function of musical texture and performers’ roles as melody or accompanying players.

## Conclusion

Our study used automatic pose estimation and Granger Causality measures to investigate the effects of musical texture on leadership dynamics reflected in instrumentalists’ head motion in videos of live concert performances of ensemble music. Findings indicate that structural features related to the hierarchical relationship between instrumental parts that vary in musical salience influence the directionality of influence between co-performers’ head motion. We observed greater directionality of influence in homophonic musical textures characterised by distinct melody and accompaniment parts, with information flowing from the melody to accompanying players, and among accompanying players, than for polyphonic textures with less clear distinction. Musical texture thus not only influences the strength of interpersonal coupling [44], but also its directionality.

These results provide proof of principle that automatic pose estimation is sufficiently sensitive to detect subtle modulations of interpersonal coupling and intricate leader-follower dynamics in standard video recordings under naturalistic conditions. This extends work on musical leadership to real-life contexts, and opens the door to future investigations of a wider range of leadership phenomena. These include studying the dynamics of coupling directionality among accompanying performers, examining different ensemble types and musical styles, measuring motion from different body parts, and exploring the relationship between interpersonal coupling dynamics of body motion and both objective and subjective measures of the coordination of musical sounds.

While our findings are context-specific to musical performances, they offer potential insights into the dynamics of leader-follower relationships in small-group interactions. To the extent that musical ensembles operate as self-regulating teams aiming for a flawless performance under systematically structured conditions governed by objective conventions and constraints (e.g., a musical score), they present an ideal opportunity to explore nonverbal communication in group activities more generally [3]. Using video-based pose estimation to analyse large publicly available datasets of real-world performances across cultures and group sizes will maximise the potential use of music to study the communication dynamics of social groups.

## Supporting information

S1 Appendix Binomial Generalised Linear Mixed Model (GLMM) analyses and additional analyses with Bonferroni correction. (PDF)

S1 Table Table for Brahms Quintet with bar numbers. (PDF)

S2 Table Table for Borodin Quartet with bar numbers. (PDF)

S3 Table Table for Granger Causality test results in Brahms Quintet. (PDF)

S4 Table Table for Granger Causality test results in Borodin Quartet. (PDF)
