# Supplementary material for: Leadership dynamics in musical groups: Quantifying effects of musical structure on directionality of influence in concert performance videos
Source: PLoS One. 2024 Apr 3;19(4):e0300663. doi: 10.1371/journal.pone.0300663 (PMC10990194; doi:10.1371/journal.pone.0300663)
Supplement: S1 Appendix — (PDF) [file pone.0300663.s001.pdf]

Supplementary Materials

S1_Appendix

For article: Leadership Dynamics in Musical Groups: Quantifying Effects of Musical Structure on Directionality of Influence in Concert Performance Videos

Authors: Sanket Rajeev Sabharwal, Matthew Breaden, Gualtiero Volpe, Antonio Camurri, and Peter E. Keller

Binomial Generalised Linear Mixed Model (GLMM) analyses

The main article reports analyses using linear mixed-effects models (LMMs) to test for global effects of musical texture on Granger Causality (GC) values, followed by Analysis of Variance (ANOVA) to address directionality-of-influence effects related to melodic leadership. However, given that the raw data consist of binary GC values (0, 1) that are not normally distributed, we also conducted binomial Generalised Linear Mixed Model (GLMM) analyses on these values to check whether equivalent effects are obtained. In this section, we report these GLMM analyses, which were run using the 'glmer' function from the lme4 (version 1.1-31) package in R (version 4.2.2) within RStudio (version 2022.12.0+353). A separate GLMM analysis addressed each of our two hypotheses.

The first GLMM analysis tested the hypothesis that there would be higher directionality in interpersonal coupling in homophonic textures (with a clear melodic leader) than in polyphonic textures (with distributed/changing leadership roles). The GLMM included texture as a fixed factor and piece, part, phrase, direction of the GC test (within each pair of instrumentalists), and instrumentalist pair (coded as unique combinations of instrumentalist numbers) as random effects. Note that instrumentalist pair is included in the GLMM but not in the LMMs in the main article because the binary values were averaged across instrumentalist pairs to get the proportion measure in the latter.

A likelihood-ratio test indicated that this full model provided a better fit to the data than a reduced model that included only the random effects ($\chi 2$ (1) = 8.21, $p < .01$; Log Likelihood = -344 (full) vs -348(reduced), AIC = 702 vs 708, BIC = 734 vs 736). For the full GLMM, there was a statistically significant effect of texture on GC values (Effect Estimate = -1.510, *SE* = 0.499, *z* = -3.03, *p* = 0.0025, 95% *CI* [-2.578 -0.509]). This outcome confirms the LMM result reported in the main article.

Supplementary Materials

The second GLMM analysis examined the effects of the musical roles of the instrumentalists (melody vs accompaniment) on the directionality of coupling. As in the corresponding LMM analysis in the main article, we included four categories of direction of influence: (1) melody instrument influencing accompanying instruments (Melody on Other), (2) accompanying instruments influencing the melody instrument (Other on Melody), (3) accompanying instruments influencing other accompanying instruments (Other on Other) in homophonic textures, and (4) mixed roles in polyphonic textures. The GLMM included direction-of-influence category as a fixed factor and piece, part, phrase, direction of the GC test, and instrumentalist pair as random effects. A likelihood-ratio test indicated that this full model provided a better fit to the data than the reduced model with only random effects ($\chi2$ (3) = 15.70, $p < .01$; Log Likelihood = -340 (full) vs -348 (reduced), AIC = 698 vs 708, BIC = 740 vs 736). There was a statistically significant effect of direction-of-influence category on GC values in the full GLMM (Effect Estimate = -1.510, *SE* = 0.499, *z* = -3.03, *p* = 0.0025, 95% *CI* [-2.578 -0.509]). Again, the outcome is consistent with the LMM result in the main article.

The direction-of-influence effect was broken down using planned orthogonal contrasts. The outcomes were essentially the same as the corresponding analysis on proportional data in the main article. Raw binary GC values were significantly higher for homophonic than polyphonic textures (*z* = 3.310, *p* = .0009, 95% *CI* [1.957, 7.635]) and for melody instrument influence on others than for other instrument influence on the melody instrument (*z* = 1.972, *p* = .049, 95% *CI* [0.005, 1.642]). However, GC values for homophonic pairings including a melody player were not significantly different from values for homophonic pairings without a melody player (*z* = -1.033, *p* = .301, 95% *CI* [-1.665, 0.515]).

In sum, the outcomes of the GLMM analyses reported here are in all respects equivalent to the LMM results in the main article.
