# Supplementary material for: Leadership dynamics in musical groups: Quantifying effects of musical structure on directionality of influence in concert performance videos
Source: PLoS One. 2024 Apr 3;19(4):e0300663. doi: 10.1371/journal.pone.0300663 (PMC10990194; doi:10.1371/journal.pone.0300663)
Supplement: S1 Table — (PDF) [file pone.0300663.s002.pdf]

Supplementary Materials

S2_Table

For article: Leadership Dynamics in Musical Groups: Quantifying Effects of Musical Structure on Directionality of Influence in Concert Performance Videos

Authors: Sanket Rajeev Sabharwal, Matthew Breaden, Gualtiero Volpe, Antonio Camurri, and Peter E. Keller

Table for Brahms Quintet with bar numbers

Description: The table below indicates the bar numbers from the score corresponding to the Brahms Quintet. The "File" column indicates the specific part of Brahms's work being analyzed, such as "BrahmsConcertPart1," " BrahmsConcertPart1" and so on. The "File No." column represents a sequence number given to each analyzed segment within the specified part. The "Start Bar" and "End Bar" columns show the range of musical bars included in each analyzed segment, essentially telling us where in the composition the segment begins and ends. The columns "Start Time" and "End Time" indicate the timings in seconds, at which the respective musical bars begin and end. The "Duration Difference" column calculates the time span of each segment by subtracting the "Start Time" from the "End Time," giving a clear picture of how long each segment lasts. The "Number of Instruments" column indicates how many instruments are being played in each segment, contributing to the understanding of the complexity of the musical composition. The "Texture" column provides insights into the texture of the musical piece. In this case, 'P' stands for 'Polyphonic' and 'H' stands for 'Homophonic'. The "Melody Instrument" column identifies the instrument playing the main melody in each segment. The abbreviations correspond to specific instruments, for instance, 'Cl' represents the clarinet, and 'Vln1' represents the first violin. In segments where the melody is shared or alternates between two instruments, both are listed, separated by a slash.

Supplementary Materials

File	File No.	Start Bar	End Bar	Start Time	End Time	Duration Difference	No. of Instruments	Texture	Melody Instrument
BrahmsConcertPart1	1	5	12	12.66	31.33	18.670	5	P	Cl
BrahmsConcertPart1	3	37	47	96.97	124.67	27.702	5	H	Cl
BrahmsConcertPart1	4	51	57	132.36	153.07	20.710	5	H	Cl
BrahmsConcertPart1	5	5	12	184.03	201.87	17.837	5	P	Cl
BrahmsConcertPart1	7	37	47	267.25	295.24	27.991	5	H	Cl
BrahmsConcertPart1	8	51	57	304.25	324.77	20.519	5	H	Cl
BrahmsConcertPart1	10	87	97	397.70	425.01	27.312	5	P	Cl/Vln1
BrahmsConcertPart1	11	98	105	425.01	448.63	23.624	5	H	Cl/Vln1
BrahmsConcertPart1	13	114	120	472.38	491.91	19.537	5	P	Cl
BrahmsConcertPart1	16	162	168	599.76	616.91	17.151	5	P	Cl/Vln1
BrahmsConcertPart1	17	172	178	625.77	646.55	20.785	5	P	Vln1
BrahmsConcertPart1	19	199	205	694.02	710.19	16.161	5	H	Vln1
BrahmsConcertPart1	20	211	218	724.92	755.35	30.433	5	H	Cl
BrahmsConcertPart2	3	27	31	84.50	103.72	19.213	5	H	Cl
BrahmsConcertPart2	7	54	56	191.64	217.04	25.392	5	P	Cl
BrahmsConcertPart2	8	57	59	217.04	235.76	18.720	5	H	Cl
BrahmsConcertPart2	9	60	62	235.76	259.39	23.633	5	P	Cl
BrahmsConcertPart2	11	68	71	288.78	313.03	24.248	5	P	Cl
BrahmsConcertPart2	13	79	87	350.75	388.95	38.199	5	H	Cl/Vln1
BrahmsConcertPart2	14	88	93	388.95	416.50	27.553	5	P	Cl
BrahmsConcertPart2	17	113	117	489.83	508.93	19.102	5	H	Cl
BrahmsConcertPart3	3	121	128	83.78	99.27	15.488	5	P	Vln1/Cl
BrahmsConcertPart3	5	153	160	171.16	186.82	15.656	5	P	Vln1/Cl
BrahmsConcertPart3	7	218	222	310.91	333.40	22.488	5	H	Cl
