# Supplementary material for: Leadership dynamics in musical groups: Quantifying effects of musical structure on directionality of influence in concert performance videos
Source: PLoS One. 2024 Apr 3;19(4):e0300663. doi: 10.1371/journal.pone.0300663 (PMC10990194; doi:10.1371/journal.pone.0300663)
Supplement: S2 Table — (PDF) [file pone.0300663.s003.pdf]

Supplementary Materials

S3_Table

For article: Leadership Dynamics in Musical Groups: Quantifying Effects of Musical Structure on Directionality of Influence in Concert Performance Videos

Authors: Sanket Rajeev Sabharwal, Matthew Breaden, Gualtiero Volpe, Antonio Camurri, and Peter E. Keller

Table for Borodin Quartet with bar numbers

Description: The table below indicates the bar numbers from the score corresponding to the Borodin Quartet. The "File" column indicates the specific part of Borodin's work being analyzed, such as "Borodin Part 1," "Borodin Part 2," and so on. The "File No." column represents a sequence number given to each analyzed segment within the specified part. The "Start Bar" and "End Bar" columns show the range of musical bars included in each analyzed segment, essentially telling us where in the composition the segment begins and ends. The columns "Start Time" and "End Time" indicate the timings in seconds, at which the respective musical bars begin and end. The "Duration Difference" column calculates the time span of each segment by subtracting the "Start Time" from the "End Time," giving a clear picture of how long each segment lasts. The "Number of Instruments" column indicates how many instruments are being played in each segment, contributing to the understanding of the complexity of the musical composition. The "Texture" column provides insights into the texture of the musical piece. In this case, 'P' stands for 'Polyphonic' and 'H' stands for 'Homophonic'. The "Melody Instrument" column identifies the instrument playing the main melody in each segment. 'NA' indicates that there isn't a single main melody instrument but rather is rather distributed. Other abbreviations correspond to specific instruments, such as 'Vln1' for first violin, 'Vlc' for cello, 'Vla' for viola, and 'Vln2' for second violin.

Supplementary Materials

File	File No.	Start Bar	End Bar	Start Time	End Time	Duration Difference	Number of Instruments	Texture	Melody Instrument
Borodin Part 1	1	9	21	13.099	34.37	21.271	4	P	NA
Borodin Part 1	2	43	56	61.962	82.539	20.577	4	P	NA
Borodin Part 2	1	380	395	262.474	277.616	15.142	4	P	NA
Borodin Part 2	2	540	553	404.765	422.774	18.009	4	H	Vln1
Borodin Part 2	3	554	569	422.774	441.398	18.624	4	H	Vln1
Borodin Part 2	4	23	32	828.574	858.202	29.628	4	P	Vln1
Borodin Part 2	5	33	40	858.202	881.197	22.995	4	P	Vlc
Borodin Part 2	6	55	62	921.932	945.796	23.864	4	H	Vln1
Borodin Part 2	7	76	82	984.981	1004.932	19.951	4	P	NA
Borodin Part 2	8	97	103	1045.337	1066.676	21.339	4	P	NA
Borodin Part 2	9	104	108	1066.676	1081.865	15.189	4	P	NA
Borodin Part 3	1	118	125	19.706	45.783	26.077	4	P	NA
Borodin Part 3	2	126	131	45.783	61.591	15.808	4	H	Vln1
Borodin Part 4	1	163	168	28.183	46.895	18.712	4	H	Vln1
Borodin Part 4	2	169	176	46.895	71.868	24.973	4	H	Vln1
Borodin Part 4	3	316	324	258.159	273.454	15.295	4	H	Vln1
Borodin Part 4	4	325	334	273.454	290.12	16.666	4	H	Vla
Borodin Part 4	5	351	360	316.721	333.126	16.405	4	H	Vln2
Borodin Part 4	6	67	79	637.503	660.318	22.815	4	H	Vln1
Borodin Part 4	7	155	164	782.907	798.735	15.828	4	P	Vln1
