# Supplementary material for: Leadership dynamics in musical groups: Quantifying effects of musical structure on directionality of influence in concert performance videos
Source: PLoS One. 2024 Apr 3;19(4):e0300663. doi: 10.1371/journal.pone.0300663 (PMC10990194; doi:10.1371/journal.pone.0300663)
Supplement: S3 Table — (PDF) [file pone.0300663.s004.pdf]

Supplementary Materials

S4_Table

For article: Leadership Dynamics in Musical Groups: Quantifying Effects of Musical Structure on Directionality of Influence in Concert Performance Videos

Authors: Sanket Rajeev Sabharwal, Matthew Breaden, Gualtiero Volpe, Antonio Camurri, and Peter E. Keller

Table for Granger Causality test results in Brahms Quintet

Description: The table below presents the results of the Granger Causality tests carried out on different parts of the Brahms Quintet. The "Part" column identifies the specific section of the Quintet being analysed. The "File No." column provides a sequential numbering of the analysed segments within each part. The "M1" and "M2" columns represent the first and second musicians in a dyadic pair being analysed. The "T" column signifies the texture of the musical piece, with 'P' representing 'Polyphonic' and 'H' denoting 'Homophonic'. The "Pair" column shows the dyadic pairs of musicians being examined for their Granger causality, such as "m1_m2", "m1_m3", etc. The "F_M1_M2" and "F_M2_M1" columns provide the F values, which are statistical measures indicating the strength of causality from musician 1 to musician 2, and vice versa. The "p_M1_M2" and "p_M2_M1" columns present the corresponding p-values, which are probabilities used to determine the significance of the observed F values. The columns "p_M1_M2 (B)" and "p_M2_M1 (B)" hold binary values (1 or 0) indicating whether the pairs Granger causes each other or not, with 1 implying causality and 0 suggesting no causality. The "M_I" column signifies the main instrument involved in the Granger Causality analysis, providing additional details about the musicians' dyadic pair and the kind of instruments they played in each analysed segment. If a combination of instruments was involved, it is represented as "Mixed".

Supplementary Materials

Part	FileNo	M1	M2	T	Pair	F_M1_M2	p_M1_M2	F_M2_M1	p_M2_M1	p_M1_M2 (B)	p_M2_M1 (B)	M_I
Part1	1	m1	m2	P	m1_m2	0.958	0.532	1.379	0.090	0	0	Mixed
Part1	1	m1	m3	P	m1_m3	0.648	0.926	1.132	0.291	0	0	Mixed
Part1	1	m1	m4	P	m1_m4	0.467	0.993	0.748	0.832	0	0	Mixed
Part1	1	m1	m5	P	m1_m5	0.487	0.991	0.949	0.546	0	0	Mixed
Part1	1	m2	m3	P	m2_m3	0.958	0.533	1.265	0.161	0	0	Mixed
Part1	1	m2	m4	P	m2_m4	0.982	0.495	1.328	0.118	0	0	Mixed
Part1	1	m2	m5	P	m2_m5	1.082	0.354	1.681	0.015	0	1	Mixed
Part1	1	m3	m4	P	m3_m4	1.697	0.013	1.182	0.236	1	0	Mixed
Part1	1	m3	m5	P	m3_m5	1.162	0.257	1.162	0.258	0	0	Mixed
Part1	1	m4	m5	P	m4_m5	0.600	0.955	1.173	0.245	0	0	Mixed
Part1	3	m1	m2	H	m1_m2	0.960	0.529	1.583	0.026	0	1	m5
Part1	3	m1	m3	H	m1_m3	1.359	0.097	1.670	0.014	0	1	m5
Part1	3	m1	m4	H	m1_m4	1.158	0.258	1.624	0.020	0	1	m5
Part1	3	m1	m5	H	m1_m5	1.383	0.085	0.981	0.496	0	0	m5
Part1	3	m2	m3	H	m2_m3	1.644	0.017	1.252	0.168	1	0	m5
Part1	3	m2	m4	H	m2_m4	1.392	0.081	1.878	0.003	0	1	m5
Part1	3	m2	m5	H	m2_m5	1.719	0.010	1.572	0.027	1	1	m5
Part1	3	m3	m4	H	m3_m4	1.888	0.003	1.181	0.234	1	0	m5
Part1	3	m3	m5	H	m3_m5	1.755	0.008	1.287	0.141	1	0	m5
Part1	3	m4	m5	H	m4_m5	1.436	0.063	1.085	0.347	0	0	m5
Part2	3	m1	m2	H	m1_m2	1.385	0.087	2.691	0.000	0	1	m5
Part2	3	m1	m3	H	m1_m3	1.847	0.005	2.656	0.000	1	1	m5
Part2	3	m1	m4	H	m1_m4	1.282	0.148	2.538	0.000	0	1	m5
Part2	3	m1	m5	H	m1_m5	1.056	0.389	2.567	0.000	0	1	m5
Part2	3	m2	m3	H	m2_m3	1.721	0.011	1.411	0.076	1	0	m5
Part2	3	m2	m4	H	m2_m4	1.767	0.008	1.568	0.030	1	1	m5
Part2	3	m2	m5	H	m2_m5	2.704	0.000	2.393	0.000	1	1	m5
Part2	3	m3	m4	H	m3_m4	1.626	0.021	1.628	0.021	1	1	m5
Part2	3	m3	m5	H	m3_m5	1.731	0.010	2.703	0.000	1	1	m5
Part2	3	m4	m5	H	m4_m5	2.415	0.000	2.117	0.001	1	1	m5
Part3	3	m1	m2	P	m1_m2	0.954	0.539	1.139	0.286	0	0	Mixed
Part3	3	m1	m3	P	m1_m3	0.827	0.729	2.173	0.000	0	1	Mixed
Part3	3	m1	m4	P	m1_m4	1.581	0.029	0.785	0.786	1	0	Mixed
Part3	3	m1	m5	P	m1_m5	0.805	0.759	1.455	0.061	0	0	Mixed
Part3	3	m2	m3	P	m2_m3	0.882	0.649	0.883	0.647	0	0	Mixed
Part3	3	m2	m4	P	m2_m4	1.653	0.019	1.191	0.230	1	0	Mixed
Part3	3	m2	m5	P	m2_m5	1.373	0.096	1.967	0.002	0	1	Mixed
Part3	3	m3	m4	P	m3_m4	1.465	0.058	0.811	0.751	0	0	Mixed
Part3	3	m3	m5	P	m3_m5	0.861	0.680	0.817	0.743	0	0	Mixed
Part3	3	m4	m5	P	m4_m5	0.709	0.873	1.085	0.351	0	0	Mixed
Part1	4	m1	m2	H	m1_m2	0.892	0.634	2.371	0.000	0	1	m5
Part1	4	m1	m3	H	m1_m3	1.346	0.107	1.184	0.233	0	0	m5
Part1	4	m1	m4	H	m1_m4	1.457	0.058	1.293	0.140	0	0	m5
Part1	4	m1	m5	H	m1_m5	0.914	0.601	2.426	0.000	0	1	m5
Part1	4	m2	m3	H	m2_m3	1.617	0.022	1.258	0.166	1	0	m5
Part1	4	m2	m4	H	m2_m4	0.697	0.887	1.984	0.002	0	1	m5
Part1	4	m2	m5	H	m2_m5	0.925	0.583	2.261	0.000	0	1	m5
Part1	4	m3	m4	H	m3_m4	1.283	0.147	1.760	0.008	0	1	m5

Supplementary Materials

Part1	4	m3	m5	H	m3_m5	0.495	0.990	0.734	0.849	0	0	m5
Part1	4	m4	m5	H	m4_m5	0.504	0.988	1.335	0.113	0	0	m5
Part1	5	m1	m2	P	m1_m2	3.013	0.000	1.444	0.063	1	0	Mixed
Part1	5	m1	m3	P	m1_m3	1.334	0.115	2.194	0.000	0	1	Mixed
Part1	5	m1	m4	P	m1_m4	1.249	0.175	1.020	0.439	0	0	Mixed
Part1	5	m1	m5	P	m1_m5	1.511	0.043	1.775	0.008	1	1	Mixed
Part1	5	m2	m3	P	m2_m3	0.661	0.916	2.907	0.000	0	1	Mixed
Part1	5	m2	m4	P	m2_m4	1.587	0.027	1.036	0.416	1	0	Mixed
Part1	5	m2	m5	P	m2_m5	3.682	0.000	3.095	0.000	1	1	Mixed
Part1	5	m3	m4	P	m3_m4	1.925	0.003	0.857	0.686	1	0	Mixed
Part1	5	m3	m5	P	m3_m5	1.864	0.004	0.945	0.553	1	0	Mixed
Part1	5	m4	m5	P	m4_m5	1.486	0.050	2.934	0.000	0	1	Mixed
Part3	5	m1	m2	P	m1_m2	1.257	0.170	1.580	0.029	0	1	Mixed
Part3	5	m1	m3	P	m1_m3	1.735	0.011	1.773	0.009	1	1	Mixed
Part3	5	m1	m4	P	m1_m4	1.631	0.021	1.223	0.199	1	0	Mixed
Part3	5	m1	m5	P	m1_m5	1.106	0.324	1.783	0.008	0	1	Mixed
Part3	5	m2	m3	P	m2_m3	1.670	0.017	0.882	0.649	1	0	Mixed
Part3	5	m2	m4	P	m2_m4	1.095	0.338	1.279	0.153	0	0	Mixed
Part3	5	m2	m5	P	m2_m5	1.149	0.273	1.022	0.437	0	0	Mixed
Part3	5	m3	m4	P	m3_m4	0.998	0.473	1.450	0.063	0	0	Mixed
Part3	5	m3	m5	P	m3_m5	1.080	0.358	1.570	0.031	0	1	Mixed
Part3	5	m4	m5	P	m4_m5	1.620	0.023	1.055	0.391	1	0	Mixed
Part1	7	m1	m2	H	m1_m2	0.511	0.987	0.710	0.875	0	0	m5
Part1	7	m1	m3	H	m1_m3	0.983	0.493	0.880	0.654	0	0	m5
Part1	7	m1	m4	H	m1_m4	0.512	0.987	1.603	0.022	0	1	m5
Part1	7	m1	m5	H	m1_m5	0.598	0.958	1.745	0.009	0	1	m5
Part1	7	m2	m3	H	m2_m3	0.884	0.647	1.266	0.157	0	0	m5
Part1	7	m2	m4	H	m2_m4	1.142	0.276	1.306	0.128	0	0	m5
Part1	7	m2	m5	H	m2_m5	0.504	0.988	0.925	0.583	0	0	m5
Part1	7	m3	m4	H	m3_m4	0.945	0.552	1.408	0.074	0	0	m5
Part1	7	m3	m5	H	m3_m5	0.785	0.789	1.565	0.029	0	1	m5
Part1	7	m4	m5	H	m4_m5	1.124	0.297	0.934	0.569	0	0	m5
Part2	7	m1	m2	P	m1_m2	1.340	0.108	0.876	0.659	0	0	Mixed
Part2	7	m1	m3	P	m1_m3	0.866	0.674	0.786	0.788	0	0	Mixed
Part2	7	m1	m4	P	m1_m4	0.684	0.899	0.899	0.624	0	0	Mixed
Part2	7	m1	m5	P	m1_m5	1.307	0.128	1.113	0.312	0	0	Mixed
Part2	7	m2	m3	P	m2_m3	1.429	0.066	0.945	0.552	0	0	Mixed
Part2	7	m2	m4	P	m2_m4	1.084	0.349	0.644	0.930	0	0	Mixed
Part2	7	m2	m5	P	m2_m5	0.907	0.611	1.007	0.457	0	0	Mixed
Part2	7	m3	m4	P	m3_m4	1.280	0.147	0.978	0.501	0	0	Mixed
Part2	7	m3	m5	P	m3_m5	0.917	0.596	0.841	0.711	0	0	Mixed
Part2	7	m4	m5	P	m4_m5	1.414	0.072	1.147	0.271	0	0	Mixed
Part3	7	m1	m2	H	m1_m2	1.838	0.005	2.273	0.000	1	1	m5
Part3	7	m1	m3	H	m1_m3	1.022	0.436	1.828	0.005	0	1	m5
Part3	7	m1	m4	H	m1_m4	3.119	0.000	0.851	0.696	1	0	m5
Part3	7	m1	m5	H	m1_m5	1.155	0.263	1.293	0.139	0	0	m5
Part3	7	m2	m3	H	m2_m3	1.323	0.119	1.799	0.006	0	1	m5
Part3	7	m2	m4	H	m2_m4	1.877	0.004	0.975	0.507	1	0	m5
Part3	7	m2	m5	H	m2_m5	2.019	0.001	1.865	0.004	1	1	m5

Supplementary Materials

Part3	7	m3	m4	H	m3_m4	0.851	0.696	0.409	0.998	0	0	m5
Part3	7	m3	m5	H	m3_m5	0.737	0.845	0.886	0.644	0	0	m5
Part3	7	m4	m5	H	m4_m5	2.197	0.000	2.223	0.000	1	1	m5
Part1	8	m1	m2	H	m1_m2	0.972	0.510	1.562	0.031	0	1	m5
Part1	8	m1	m3	H	m1_m3	1.873	0.004	1.960	0.002	1	1	m5
Part1	8	m1	m4	H	m1_m4	2.866	0.000	1.925	0.003	1	1	m5
Part1	8	m1	m5	H	m1_m5	3.205	0.000	6.885	0.000	1	1	m5
Part1	8	m2	m3	H	m2_m3	1.024	0.433	1.251	0.172	0	0	m5
Part1	8	m2	m4	H	m2_m4	1.519	0.040	1.124	0.300	1	0	m5
Part1	8	m2	m5	H	m2_m5	1.697	0.013	2.083	0.001	1	1	m5
Part1	8	m3	m4	H	m3_m4	5.800	0.000	2.186	0.000	1	1	m5
Part1	8	m3	m5	H	m3_m5	4.770	0.000	1.349	0.105	1	0	m5
Part1	8	m4	m5	H	m4_m5	3.880	0.000	1.792	0.007	1	1	m5
Part2	8	m1	m2	H	m1_m2	1.270	0.158	0.790	0.781	0	0	m5
Part2	8	m1	m3	H	m1_m3	0.694	0.888	1.547	0.034	0	1	m5
Part2	8	m1	m4	H	m1_m4	0.912	0.604	1.037	0.415	0	0	m5
Part2	8	m1	m5	H	m1_m5	0.809	0.755	0.748	0.832	0	0	m5
Part2	8	m2	m3	H	m2_m3	0.942	0.557	0.889	0.638	0	0	m5
Part2	8	m2	m4	H	m2_m4	1.186	0.232	1.065	0.376	0	0	m5
Part2	8	m2	m5	H	m2_m5	0.784	0.789	1.334	0.115	0	0	m5
Part2	8	m3	m4	H	m3_m4	1.203	0.216	0.593	0.959	0	0	m5
Part2	8	m3	m5	H	m3_m5	0.888	0.640	1.149	0.271	0	0	m5
Part2	8	m4	m5	H	m4_m5	0.685	0.897	0.907	0.611	0	0	m5
Part2	9	m1	m2	P	m1_m2	1.743	0.009	1.259	0.163	1	0	Mixed
Part2	9	m1	m3	P	m1_m3	0.947	0.549	0.777	0.798	0	0	Mixed
Part2	9	m1	m4	P	m1_m4	0.825	0.734	1.148	0.270	0	0	Mixed
Part2	9	m1	m5	P	m1_m5	1.003	0.463	1.000	0.468	0	0	Mixed
Part2	9	m2	m3	P	m2_m3	1.075	0.361	1.398	0.079	0	0	Mixed
Part2	9	m2	m4	P	m2_m4	0.891	0.636	1.304	0.131	0	0	Mixed
Part2	9	m2	m5	P	m2_m5	0.861	0.681	0.664	0.915	0	0	Mixed
Part2	9	m3	m4	P	m3_m4	1.198	0.217	0.947	0.549	0	0	Mixed
Part2	9	m3	m5	P	m3_m5	1.266	0.158	1.370	0.092	0	0	Mixed
Part2	9	m4	m5	P	m4_m5	1.101	0.327	0.861	0.681	0	0	Mixed
Part1	10	m1	m2	P	m1_m2	0.915	0.598	1.785	0.007	0	1	Mixed
Part1	10	m1	m3	P	m1_m3	0.513	0.986	0.958	0.531	0	0	Mixed
Part1	10	m1	m4	P	m1_m4	1.495	0.044	0.985	0.491	1	0	Mixed
Part1	10	m1	m5	P	m1_m5	2.446	0.000	1.255	0.165	1	0	Mixed
Part1	10	m2	m3	P	m2_m3	0.969	0.514	0.680	0.903	0	0	Mixed
Part1	10	m2	m4	P	m2_m4	0.643	0.931	0.717	0.868	0	0	Mixed
Part1	10	m2	m5	P	m2_m5	1.270	0.154	0.802	0.767	0	0	Mixed
Part1	10	m3	m4	P	m3_m4	0.957	0.533	0.724	0.861	0	0	Mixed
Part1	10	m3	m5	P	m3_m5	1.023	0.434	1.251	0.169	0	0	Mixed
Part1	10	m4	m5	P	m4_m5	1.149	0.268	1.001	0.467	0	0	Mixed
Part1	11	m1	m2	H	m1_m2	1.254	0.168	3.225	0.000	0	1	m5
Part1	11	m1	m3	H	m1_m3	1.440	0.062	3.392	0.000	0	1	m5
Part1	11	m1	m4	H	m1_m4	1.482	0.049	3.710	0.000	1	1	m5
Part1	11	m1	m5	H	m1_m5	1.471	0.052	3.057	0.000	0	1	m5
Part1	11	m2	m3	H	m2_m3	1.845	0.004	5.165	0.000	1	1	m5
Part1	11	m2	m4	H	m2_m4	1.065	0.375	4.080	0.000	0	1	m5

Supplementary Materials

Part1	11	m2	m5	H	m2_m5	2.708	0.000	3.838	0.000	1	1	m5
Part1	11	m3	m4	H	m3_m4	2.656	0.000	1.596	0.024	1	1	m5
Part1	11	m3	m5	H	m3_m5	4.195	0.000	1.725	0.010	1	1	m5
Part1	11	m4	m5	H	m4_m5	4.692	0.000	2.191	0.000	1	1	m5
Part2	11	m1	m2	P	m1_m2	1.705	0.012	1.074	0.362	1	0	Mixed
Part2	11	m1	m3	P	m1_m3	1.070	0.368	1.558	0.031	0	1	Mixed
Part2	11	m1	m4	P	m1_m4	1.154	0.263	1.064	0.375	0	0	Mixed
Part2	11	m1	m5	P	m1_m5	1.065	0.375	1.261	0.162	0	0	Mixed
Part2	11	m2	m3	P	m2_m3	0.718	0.866	0.934	0.569	0	0	Mixed
Part2	11	m2	m4	P	m2_m4	1.590	0.025	1.095	0.334	1	0	Mixed
Part2	11	m2	m5	P	m2_m5	1.138	0.282	0.624	0.943	0	0	Mixed
Part2	11	m3	m4	P	m3_m4	1.585	0.026	0.989	0.484	1	0	Mixed
Part2	11	m3	m5	P	m3_m5	0.923	0.586	1.235	0.183	0	0	Mixed
Part2	11	m4	m5	P	m4_m5	1.500	0.044	1.302	0.132	1	0	Mixed
Part1	13	m1	m2	P	m1_m2	1.059	0.383	2.552	0.000	0	1	Mixed
Part1	13	m1	m3	P	m1_m3	1.431	0.067	1.450	0.061	0	0	Mixed
Part1	13	m1	m4	P	m1_m4	2.208	0.000	1.439	0.064	1	0	Mixed
Part1	13	m1	m5	P	m1_m5	0.440	0.996	1.670	0.016	0	1	Mixed
Part1	13	m2	m3	P	m2_m3	2.127	0.001	0.468	0.993	1	0	Mixed
Part1	13	m2	m4	P	m2_m4	3.062	0.000	1.125	0.299	1	0	Mixed
Part1	13	m2	m5	P	m2_m5	1.724	0.011	1.471	0.054	1	0	Mixed
Part1	13	m3	m4	P	m3_m4	2.158	0.000	0.989	0.485	1	0	Mixed
Part1	13	m3	m5	P	m3_m5	0.844	0.706	1.996	0.002	0	1	Mixed
Part1	13	m4	m5	P	m4_m5	1.502	0.045	3.496	0.000	1	1	Mixed
Part2	13	m1	m2	H	m1_m2	1.275	0.148	1.207	0.205	0	0	m5
Part2	13	m1	m3	H	m1_m3	1.137	0.280	1.234	0.181	0	0	m5
Part2	13	m1	m4	H	m1_m4	1.510	0.039	0.942	0.557	1	0	m5
Part2	13	m1	m5	H	m1_m5	2.018	0.001	1.431	0.063	1	0	m5
Part2	13	m2	m3	H	m2_m3	0.768	0.811	0.768	0.812	0	0	m5
Part2	13	m2	m4	H	m2_m4	1.184	0.229	1.139	0.278	0	0	m5
Part2	13	m2	m5	H	m2_m5	0.597	0.959	1.159	0.256	0	0	m5
Part2	13	m3	m4	H	m3_m4	0.904	0.617	0.881	0.652	0	0	m5
Part2	13	m3	m5	H	m3_m5	0.930	0.576	0.957	0.533	0	0	m5
Part2	13	m4	m5	H	m4_m5	1.076	0.358	1.123	0.297	0	0	m5
Part2	14	m1	m2	P	m1_m2	0.716	0.869	0.863	0.679	0	0	Mixed
Part2	14	m1	m3	P	m1_m3	1.126	0.295	0.604	0.954	0	0	Mixed
Part2	14	m1	m4	P	m1_m4	1.651	0.016	1.014	0.447	1	0	Mixed
Part2	14	m1	m5	P	m1_m5	0.969	0.514	0.914	0.601	0	0	Mixed
Part2	14	m2	m3	P	m2_m3	0.957	0.534	0.773	0.804	0	0	Mixed
Part2	14	m2	m4	P	m2_m4	0.618	0.947	1.020	0.438	0	0	Mixed
Part2	14	m2	m5	P	m2_m5	0.522	0.984	1.223	0.193	0	0	Mixed
Part2	14	m3	m4	P	m3_m4	0.458	0.995	0.537	0.981	0	0	Mixed
Part2	14	m3	m5	P	m3_m5	0.717	0.868	0.520	0.985	0	0	Mixed
Part2	14	m4	m5	P	m4_m5	1.367	0.093	1.121	0.301	0	0	Mixed
Part1	16	m1	m2	P	m1_m2	1.097	0.334	1.579	0.029	0	1	Mixed
Part1	16	m1	m3	P	m1_m3	1.391	0.086	0.861	0.681	0	0	Mixed
Part1	16	m1	m4	P	m1_m4	0.732	0.850	1.019	0.442	0	0	Mixed
Part1	16	m1	m5	P	m1_m5	0.775	0.799	1.599	0.025	0	1	Mixed
Part1	16	m2	m3	P	m2_m3	0.828	0.729	0.712	0.871	0	0	Mixed

Supplementary Materials

Part1	16	m2	m4	P	m2_m4	0.913	0.602	0.984	0.493	0	0	Mixed
Part1	16	m2	m5	P	m2_m5	1.082	0.353	1.509	0.044	0	1	Mixed
Part1	16	m3	m4	P	m3_m4	1.072	0.368	1.172	0.247	0	0	Mixed
Part1	16	m3	m5	P	m3_m5	1.302	0.135	1.238	0.185	0	0	Mixed
Part1	16	m4	m5	P	m4_m5	0.854	0.690	1.020	0.440	0	0	Mixed
Part1	17	m1	m2	P	m1_m2	1.506	0.043	1.327	0.118	1	0	Mixed
Part1	17	m1	m3	P	m1_m3	1.306	0.130	1.024	0.432	0	0	Mixed
Part1	17	m1	m4	P	m1_m4	0.886	0.644	1.117	0.308	0	0	Mixed
Part1	17	m1	m5	P	m1_m5	1.375	0.091	1.204	0.213	0	0	Mixed
Part1	17	m2	m3	P	m2_m3	1.230	0.189	1.423	0.070	0	0	Mixed
Part1	17	m2	m4	P	m2_m4	1.228	0.191	0.899	0.623	0	0	Mixed
Part1	17	m2	m5	P	m2_m5	1.205	0.212	0.881	0.652	0	0	Mixed
Part1	17	m3	m4	P	m3_m4	1.182	0.235	1.416	0.073	0	0	Mixed
Part1	17	m3	m5	P	m3_m5	1.172	0.245	0.982	0.495	0	0	Mixed
Part1	17	m4	m5	P	m4_m5	1.069	0.369	1.670	0.015	0	1	Mixed
Part2	17	m1	m2	H	m1_m2	1.602	0.024	1.299	0.137	1	0	m5
Part2	17	m1	m3	H	m1_m3	1.458	0.058	1.689	0.014	0	1	m5
Part2	17	m1	m4	H	m1_m4	3.051	0.000	1.291	0.142	1	0	m5
Part2	17	m1	m5	H	m1_m5	1.267	0.160	1.197	0.220	0	0	m5
Part2	17	m2	m3	H	m2_m3	1.698	0.013	2.454	0.000	1	1	m5
Part2	17	m2	m4	H	m2_m4	2.352	0.000	1.840	0.005	1	1	m5
Part2	17	m2	m5	H	m2_m5	1.486	0.050	2.228	0.000	1	1	m5
Part2	17	m3	m4	H	m3_m4	3.208	0.000	1.817	0.006	1	1	m5
Part2	17	m3	m5	H	m3_m5	1.780	0.008	2.474	0.000	1	1	m5
Part2	17	m4	m5	H	m4_m5	2.032	0.001	6.557	0.000	1	1	m5
Part1	19	m1	m2	P	m1_m2	1.038	0.414	1.325	0.121	0	0	m1
Part1	19	m1	m3	P	m1_m3	0.966	0.520	1.311	0.131	0	0	m1
Part1	19	m1	m4	P	m1_m4	1.812	0.006	1.486	0.051	1	0	m1
Part1	19	m1	m5	P	m1_m5	1.719	0.012	1.361	0.101	1	0	m1
Part1	19	m2	m3	P	m2_m3	1.646	0.019	3.502	0.000	1	1	m1
Part1	19	m2	m4	P	m2_m4	1.802	0.007	2.029	0.001	1	1	m1
Part1	19	m2	m5	P	m2_m5	1.666	0.017	1.455	0.060	1	0	m1
Part1	19	m3	m4	P	m3_m4	1.799	0.007	1.766	0.009	1	1	m1
Part1	19	m3	m5	P	m3_m5	1.875	0.004	1.774	0.008	1	1	m1
Part1	19	m4	m5	P	m4_m5	1.205	0.214	1.621	0.022	0	1	m1
Part1	20	m1	m2	H	m1_m2	1.700	0.012	1.142	0.276	1	0	m5
Part1	20	m1	m3	H	m1_m3	1.074	0.362	1.676	0.014	0	1	m5
Part1	20	m1	m4	H	m1_m4	1.362	0.095	2.059	0.001	0	1	m5
Part1	20	m1	m5	H	m1_m5	1.559	0.029	1.947	0.002	1	1	m5
Part1	20	m2	m3	H	m2_m3	1.063	0.376	1.800	0.006	0	1	m5
Part1	20	m2	m4	H	m2_m4	1.308	0.126	2.111	0.001	0	1	m5
Part1	20	m2	m5	H	m2_m5	0.799	0.771	1.818	0.005	0	1	m5
Part1	20	m3	m4	H	m3_m4	1.479	0.048	1.610	0.021	1	1	m5
Part1	20	m3	m5	H	m3_m5	1.642	0.017	2.056	0.001	1	1	m5
Part1	20	m4	m5	H	m4_m5	1.862	0.004	1.377	0.087	1	0	m5
