# Supplementary material for: Leadership dynamics in musical groups: Quantifying effects of musical structure on directionality of influence in concert performance videos
Source: PLoS One. 2024 Apr 3;19(4):e0300663. doi: 10.1371/journal.pone.0300663 (PMC10990194; doi:10.1371/journal.pone.0300663)
Supplement: S4 Table — (PDF) [file pone.0300663.s005.pdf]

Supplementary Materials

S5_Table

For article: Leadership Dynamics in Musical Groups: Quantifying Effects of Musical Structure on Directionality of Influence in Concert Performance Videos

Authors: Sanket Rajeev Sabharwal, Matthew Breaden, Gualtiero Volpe, Antonio Camurri, and Peter E. Keller

Table for Granger Causality test results in Borodin Quartet

Description: The table below presents the results of the Granger Causality tests carried out on different parts of the Borodin Quartet. The "Part" column identifies the specific section of the Quartet being analyzed. The "File No." column provides a sequential numbering of the analyzed segments within each part. The "M1" and "M2" columns represent the first and second musicians in a dyadic pair being analyzed. The "T" column signifies the texture of the musical piece, with 'P' representing 'Polyphonic' and 'H' denoting 'Homophonic'. The "Pair" column shows the dyadic pairs of musicians being examined for their Granger causality, such as "m1_m2", "m1_m3", etc. The "F_M1_M2" and "F_M2_M1" columns provide the F values, which are statistical measures indicating the strength of causality from musician 1 to musician 2, and vice versa. The "p_M1_M2" and "p_M2_M1" columns present the corresponding p-values, which are probabilities used to determine the significance of the observed F values. The columns "p_M1_M2 (B)" and "p_M2_M1 (B)" hold binary values (1 or 0) indicating whether the pairs Granger causes each other or not, with 1 implying causality and 0 suggesting no causality. The "M_I" column signifies the main instrument involved in the Granger Causality analysis, providing additional details about the musicians' dyadic pair and the kind of instruments they played in each analysed segment. If a combination of instruments was involved, it is represented as "Mixed".

Supplementary Materials

Part	FileNo	M1	M2	T	Pair	F_M1_M2	p_M1_M2	F_M2_M1	p_M2_M1	p_M1_M2 (B)	p_M2_M1 (B)	M_I
Part1	1	m1	m2	P	m1_m2	1.185	0.231	1.311	0.127	0	0	Mixed
Part1	1	m1	m3	P	m1_m3	0.622	0.944	0.692	0.891	0	0	Mixed
Part1	1	m1	m4	P	m1_m4	0.913	0.602	1.063	0.378	0	0	Mixed
Part1	1	m2	m3	P	m2_m3	1.035	0.418	1.041	0.409	0	0	Mixed
Part1	1	m2	m4	P	m2_m4	1.476	0.051	0.885	0.646	0	0	Mixed
Part1	1	m3	m4	P	m3_m4	0.588	0.962	1.181	0.235	0	0	Mixed
Part1	2	m1	m2	P	m1_m2	1.259	0.165	1.152	0.267	0	0	Mixed
Part1	2	m1	m3	P	m1_m3	0.771	0.806	1.049	0.397	0	0	Mixed
Part1	2	m1	m4	P	m1_m4	1.239	0.182	0.825	0.733	0	0	Mixed
Part1	2	m2	m3	P	m2_m3	1.104	0.324	0.814	0.749	0	0	Mixed
Part1	2	m2	m4	P	m2_m4	0.805	0.761	0.839	0.714	0	0	Mixed
Part1	2	m3	m4	P	m3_m4	1.360	0.099	1.437	0.065	0	0	Mixed
Part2	1	m1	m2	P	m1_m2	0.712	0.869	0.827	0.729	0	0	Mixed
Part2	1	m1	m3	P	m1_m3	0.760	0.817	0.702	0.879	0	0	Mixed
Part2	1	m1	m4	P	m1_m4	1.761	0.009	1.486	0.052	1	0	Mixed
Part2	1	m2	m3	P	m2_m3	0.767	0.808	0.988	0.488	0	0	Mixed
Part2	1	m2	m4	P	m2_m4	1.456	0.061	0.827	0.729	0	0	Mixed
Part2	1	m3	m4	P	m3_m4	1.037	0.417	0.992	0.482	0	0	Mixed
Part2	2	m1	m2	H	m1_m2	1.263	0.163	1.151	0.269	0	0	m1
Part2	2	m1	m3	H	m1_m3	0.682	0.899	1.034	0.420	0	0	m1
Part2	2	m1	m4	H	m1_m4	0.824	0.735	1.048	0.400	0	0	m1
Part2	2	m2	m3	H	m2_m3	0.869	0.669	0.765	0.812	0	0	m1
Part2	2	m2	m4	H	m2_m4	0.888	0.639	1.180	0.238	0	0	m1
Part2	2	m3	m4	H	m3_m4	1.906	0.003	1.360	0.100	1	0	m1
Part2	3	m1	m2	H	m1_m2	1.354	0.103	1.239	0.182	0	0	m1
Part2	3	m1	m3	H	m1_m3	1.377	0.091	0.993	0.479	0	0	m1
Part2	3	m1	m4	H	m1_m4	1.301	0.135	1.170	0.249	0	0	m1
Part2	3	m2	m3	H	m2_m3	1.199	0.219	1.273	0.155	0	0	m1
Part2	3	m2	m4	H	m2_m4	0.934	0.569	1.183	0.235	0	0	m1
Part2	3	m3	m4	H	m3_m4	1.062	0.380	1.238	0.184	0	0	m1
Part2	4	m1	m2	P	m1_m2	0.573	0.969	1.587	0.025	0	1	Mixed
Part2	4	m1	m3	P	m1_m3	1.151	0.266	1.307	0.127	0	0	Mixed
Part2	4	m1	m4	P	m1_m4	0.837	0.718	0.932	0.572	0	0	Mixed
Part2	4	m2	m3	P	m2_m3	0.941	0.558	0.787	0.787	0	0	Mixed
Part2	4	m2	m4	P	m2_m4	1.498	0.043	1.465	0.053	1	0	Mixed
Part2	4	m3	m4	P	m3_m4	1.139	0.280	0.935	0.568	0	0	Mixed
Part2	5	m1	m2	P	m1_m2	1.024	0.433	1.192	0.223	0	0	Mixed
Part2	5	m1	m3	P	m1_m3	0.988	0.486	0.855	0.691	0	0	Mixed
Part2	5	m1	m4	P	m1_m4	0.936	0.567	0.610	0.951	0	0	Mixed
Part2	5	m2	m3	P	m2_m3	1.005	0.461	0.748	0.834	0	0	Mixed
Part2	5	m2	m4	P	m2_m4	0.871	0.666	1.351	0.102	0	0	Mixed
Part2	5	m3	m4	P	m3_m4	1.305	0.131	1.064	0.377	0	0	Mixed
Part2	6	m1	m2	H	m1_m2	1.348	0.104	1.073	0.363	0	0	m1
Part2	6	m1	m3	H	m1_m3	0.869	0.669	1.218	0.198	0	0	m1
Part2	6	m1	m4	H	m1_m4	1.548	0.033	1.238	0.181	1	0	m1
Part2	6	m2	m3	H	m2_m3	1.094	0.335	0.690	0.893	0	0	m1
Part2	6	m2	m4	H	m2_m4	0.875	0.660	0.841	0.711	0	0	m1
Part2	6	m3	m4	H	m3_m4	0.985	0.490	0.473	0.993	0	0	m1

Supplementary Materials

Part2	7	m1	m2	P	m1_m2	1.149	0.271	1.436	0.065	0	0	Mixed
Part2	7	m1	m3	P	m1_m3	1.453	0.059	0.989	0.485	0	0	Mixed
Part2	7	m1	m4	P	m1_m4	0.659	0.918	1.201	0.216	0	0	Mixed
Part2	7	m2	m3	P	m2_m3	1.022	0.436	0.755	0.825	0	0	Mixed
Part2	7	m2	m4	P	m2_m4	1.316	0.124	0.838	0.715	0	0	Mixed
Part2	7	m3	m4	P	m3_m4	1.050	0.396	1.169	0.248	0	0	Mixed
Part2	8	m1	m2	P	m1_m2	1.481	0.050	0.930	0.576	1	0	Mixed
Part2	8	m1	m3	P	m1_m3	2.201	0.000	1.062	0.379	1	0	Mixed
Part2	8	m1	m4	P	m1_m4	1.019	0.440	1.154	0.264	0	0	Mixed
Part2	8	m2	m3	P	m2_m3	1.279	0.149	1.208	0.209	0	0	Mixed
Part2	8	m2	m4	P	m2_m4	0.984	0.492	1.274	0.153	0	0	Mixed
Part2	8	m3	m4	P	m3_m4	1.331	0.115	1.105	0.323	0	0	Mixed
Part2	9	m1	m2	P	m1_m2	0.878	0.654	1.258	0.170	0	0	Mixed
Part2	9	m1	m3	P	m1_m3	0.950	0.546	1.170	0.252	0	0	Mixed
Part2	9	m1	m4	P	m1_m4	1.269	0.162	0.887	0.641	0	0	Mixed
Part2	9	m2	m3	P	m2_m3	1.203	0.218	1.026	0.432	0	0	Mixed
Part2	9	m2	m4	P	m2_m4	1.187	0.234	2.016	0.002	0	1	Mixed
Part2	9	m3	m4	P	m3_m4	0.926	0.581	1.045	0.405	0	0	Mixed
Part3	1	m1	m2	P	m1_m2	1.313	0.124	1.072	0.365	0	0	Mixed
Part3	1	m1	m3	P	m1_m3	0.952	0.542	1.304	0.130	0	0	Mixed
Part3	1	m1	m4	P	m1_m4	1.370	0.092	1.008	0.457	0	0	Mixed
Part3	1	m2	m3	P	m2_m3	1.086	0.346	0.877	0.657	0	0	Mixed
Part3	1	m2	m4	P	m2_m4	1.421	0.069	1.065	0.374	0	0	Mixed
Part3	1	m3	m4	P	m3_m4	0.939	0.561	1.088	0.343	0	0	Mixed
Part3	2	m1	m2	H	m1_m2	4.991	0.000	2.711	0.000	1	1	m1
Part3	2	m1	m3	H	m1_m3	8.441	0.000	3.541	0.000	1	1	m1
Part3	2	m1	m4	H	m1_m4	2.302	0.000	2.760	0.000	1	1	m1
Part3	2	m2	m3	H	m2_m3	2.551	0.000	2.814	0.000	1	1	m1
Part3	2	m2	m4	H	m2_m4	2.545	0.000	5.839	0.000	1	1	m1
Part3	2	m3	m4	H	m3_m4	2.003	0.002	3.693	0.000	1	1	m1
Part4	1	m1	m2	H	m1_m2	1.149	0.271	1.300	0.136	0	0	m1
Part4	1	m1	m3	H	m1_m3	1.027	0.429	0.876	0.658	0	0	m1
Part4	1	m1	m4	H	m1_m4	1.151	0.269	1.119	0.306	0	0	m1
Part4	1	m2	m3	H	m2_m3	1.587	0.027	1.380	0.089	1	0	m1
Part4	1	m2	m4	H	m2_m4	0.665	0.913	0.584	0.963	0	0	m1
Part4	1	m3	m4	H	m3_m4	1.705	0.013	1.798	0.007	1	1	m1
Part4	2	m1	m2	H	m1_m2	0.889	0.639	0.991	0.481	0	0	m1
Part4	2	m1	m3	H	m1_m3	1.046	0.401	0.713	0.872	0	0	m1
Part4	2	m1	m4	H	m1_m4	1.124	0.299	0.652	0.925	0	0	m1
Part4	2	m2	m3	H	m2_m3	1.403	0.077	1.224	0.193	0	0	m1
Part4	2	m2	m4	H	m2_m4	1.296	0.136	0.953	0.540	0	0	m1
Part4	2	m3	m4	H	m3_m4	0.796	0.774	0.692	0.892	0	0	m1
Part4	3	m1	m2	H	m1_m2	0.788	0.782	0.638	0.932	0	0	m1
Part4	3	m1	m3	H	m1_m3	0.643	0.928	0.995	0.478	0	0	m1
Part4	3	m1	m4	H	m1_m4	0.653	0.922	0.933	0.572	0	0	m1
Part4	3	m2	m3	H	m2_m3	0.733	0.848	1.668	0.017	0	1	m1
Part4	3	m2	m4	H	m2_m4	1.200	0.221	0.416	0.998	0	0	m1
Part4	3	m3	m4	H	m3_m4	1.675	0.016	0.331	1.000	1	0	m1
Part4	4	m1	m2	H	m1_m2	1.066	0.376	1.618	0.023	0	1	m3

Part4	4	m1	m3	H	m1_m3	1.321	0.124	0.938	0.564	0	0	m3
Part4	4	m1	m4	H	m1_m4	0.534	0.980	1.415	0.075	0	0	m3
Part4	4	m2	m3	H	m2_m3	1.345	0.110	1.465	0.057	0	0	m3
Part4	4	m2	m4	H	m2_m4	1.538	0.037	2.264	0.000	1	1	m3
Part4	4	m3	m4	H	m3_m4	1.531	0.039	0.904	0.615	1	0	m3
Part4	5	m1	m2	H	m1_m2	1.176	0.244	2.464	0.000	0	1	m2
Part4	5	m1	m3	H	m1_m3	1.561	0.033	2.487	0.000	1	1	m2
Part4	5	m1	m4	H	m1_m4	1.323	0.123	1.594	0.027	0	1	m2
Part4	5	m2	m3	H	m2_m3	2.035	0.001	1.803	0.007	1	1	m2
Part4	5	m2	m4	H	m2_m4	1.597	0.026	0.991	0.483	1	0	m2
Part4	5	m3	m4	H	m3_m4	2.387	0.000	0.771	0.804	1	0	m2
Part4	6	m1	m2	H	m1_m2	0.714	0.870	0.848	0.701	0	0	m1
Part4	6	m1	m3	H	m1_m3	1.483	0.049	0.778	0.797	1	0	m1
Part4	6	m1	m4	H	m1_m4	1.172	0.245	1.161	0.256	0	0	m1
Part4	6	m2	m3	H	m2_m3	1.432	0.066	1.205	0.211	0	0	m1
Part4	6	m2	m4	H	m2_m4	1.139	0.281	0.983	0.493	0	0	m1
Part4	6	m3	m4	H	m3_m4	0.810	0.755	0.542	0.979	0	0	m1
Part4	7	m1	m2	H	m1_m2	1.189	0.231	1.686	0.015	0	1	Mixed
Part4	7	m1	m3	H	m1_m3	1.237	0.187	0.812	0.751	0	0	Mixed
Part4	7	m1	m4	H	m1_m4	0.631	0.937	0.938	0.563	0	0	Mixed
Part4	7	m2	m3	H	m2_m3	1.200	0.220	1.250	0.176	0	0	Mixed
Part4	7	m2	m4	H	m2_m4	0.655	0.920	1.413	0.077	0	0	Mixed
Part4	7	m3	m4	H	m3_m4	1.029	0.427	0.887	0.641	0	0	Mixed
